# An Historical Essay on the State of the Medical Sciences during the Last Six Months

**Published:** 1823-01

**Authors:** 


THE LONDON

### Medical and Physical Journal.

1 OF VOL. XLIX.] JANUARY, 1823. [No. 287.

For many fortunate discoveries in medicine, and for the detection of numerous errors, the world is indebted to the rapid circulation of Monthly Journals; and there never existed any work, to which the Faculty, in Europe and America, were under deeper obligations, than to the Medical and Physical Journal of London, now forming a long, but an invaluable, series.—RUSH.

### AN HISTORICAL ESSAY
ON
### THE STATE OF THE MEDICAL SCIENCES
DURING THE LAST SIX MONTHS.

ON presenting our readers with a Retrospect of the Progress of Medicine and the collateral Sciences, during the last six months, it may not be irrelevant to say a few words regarding the view we have taken of the manner in which this may be best effected. An attempt to notice every thing that has been published within this period, would be to reduce the essay to a mere catalogue of the papers contained in the various foreign and domestic Journals, and a reference to the very limited number of more extended works which have appeared. On this account, we have judged it more expedient to fix upon some of the most important subjects to which attention has been directed, and to concentrate, as much as possible, any new light which has been thrown upon them, so as to assist the memories of those who may have been progressively following us in our monthly career; and, at the same time, to point out some of the most interesting cases and occurrences of the last few months to any others who may be desirous to avail themselves of this Sketch, which may be considered as a brief medical chronicle of the times. We therefore request our readers to observe, that we do not now, nor do we intend hereafter, to attempt more than to select from the general mass of medical writings such parts as appear to us to merit, in an especial manner, the attention of our professional brethren; and we feel convinced that those best acquainted with the labour and difficulty of the task will be the most disposed to view with indulgence the deficiencies in its execution.

In pursuance of this plan, we purpose adopting the following divisions, both for the sake of perspicuity and facilitating the means of reference:—viz. ANATOMY, *natural*, *comparative*, and *morbid*; PHYSIOLOGY, PATHOLOGY, MEDICINE, THERAPEUTICS and

Materia Medica, Surgery, Midwifery, Chemistry, and Botany.

### ANATOMY (NATURAL.)

If we consider how long this branch of natural science has engaged attention, and how many gifted individuals have devoted themselves to its investigation, we must regard it as matter of astonishment that any new discovery should be left for anatomists of the present day, not wonder at the lack of novelty. Within the period of our Essay, the anatomical structure of the eye has received some elucidation from the Croonian Lecture of Sir E. Home, illustrated by the admirable microscopical drawings of Mr. Bauer.* It appears that the marsupium in the bird's eye is not muscular, but a fine vascular membrane, as supposed by Dr. Young. The anterior layer of the ciliary processes consists of about eighty processes lying directly behind the iris, and firmly attached at the base to the sclerotic and choroid coats. Between these membranous processes are little bundles of muscular fibres, $\frac{25}{100}$ of an inch long: they take their rise from the capsule of the vitreous humour, pass over the edge of the lens, and are attached to its capsule, unconnected either with the iris or ciliary processes. In the human eye, they form bundles; in birds, they form nearly one continuous layer of muscular fibres. "The iris is fixed at its origin to the annular ligament, is divisable into two layers, the posterior muscular, the fibres radiating towards the pupil, at which part there is a regular sphincter muscle; the anterior membranous." This structure had been previously described and delineated by Mr. Maunoir, of Geneva.

A new muscle has been described in the human eye, by Dr. Horner, of Philadelphia.† It takes its origin from the os unguis, near its junction with the os planum, and passes forwards and outwards to the internal commissure of the eyelids, near the puncta lachrymalia, where it terminates. Previous to this, however, it separates into two parts; one of which is inserted into the upper eyelid, and the other into the lower. The muscle is about half an inch long, and one-fourth of an inch wide, its margins being well defined. To get a view of this muscle, we are directed "to cut through the eyelids, and separate them from the ball, except at the internal canthus; then turn the lids over on the nose, remove the valvula semilunaris, and the tunica conjunctiva in the neighbourhood, with the fatty matter." Its use is to draw the puncta inwards, and to keep the eyelids properly applied to the ball.

The minute structure of particular organs still holds out a field for new discoveries; or, at least, new descriptions of them are given. Dr. J. M. Mappes,‡ of Francfort, has made the liver and kidneys the subject of a thesis, which is to be regarded rather as conveying an account of the facts detailed by Professor Autenrieth, of Tubingen,§ and an analysis of the dissertation by M. Eysenhardt,|| than as

* *Philosophical Transactions*, 1822.

† *London Medical Repository*, July 1822.

‡ *Journal Complémentaire*, Mai.

§ *De Penitiori Hepatis Humani Structura*. 4to. Tubingæ, 1817.

|| *De Structurâ Renum Observationes Anatomicæ*. 4to. Berlin, 1818.

containing any new observations peculiar to himself. If the liver, in its recent state, be examined, either externally or internally, by cutting or tearing its substance, it appears throughout composed of two different textures: one of these, which is termed *granular* by Mappes, and *medullary* by Autenreith, forms convolutions, sometimes resembling those of the intestines, sometimes branched, flattish, rounded, of a yellow colour, and considerable density, which leave between them rounded spaces, from a quarter to half a line in breadth; which spaces are filled up by a substance, which is brown, and called cellulo-vascular, or cortical, according to M. Autenreith. When the liver is much gorged with blood, these structures cannot be distinguished without previously injecting warm water into the large vessels, especially the vena portæ. Examined with the microscope, these convolutions appear entirely composed of small grains. If water, with cinnabar or other colouring matter suspended in it, be injected into a branch of the hepatic vein, the water flows out by the vena portæ, but the colouring matter is deposited on the sides of the vessels, so that, being examined with the microscope, the texture of the viscus appears divided into little brilliant granulations. Among the convolutions of this substance are little triangular openings, which communicate with each other: some of these only contain ramifications of the hepatic vein; but in others, particularly if examined at a greater depth, are to be found three vessels, —viz. a large branch which belongs to the hepatic vein, and two smaller, one of which appertains to the artery, and the other to the hepatic duct. The sides of none of the vessels which arise from trunks contained in the capsule of Glisson adhere to the intimate structure of the liver, but are separated from it by a gelatinous matter, which appears uniform; and in some degree, also, by a portion of cellular substance which accompanies them. The hepatic vein presents no similar appearance; its ramifications adhere intimately with the granular substance, without any medium of separation.

With regard to the kidney, the method employed by M. Eysenhardt was to take very thin slices, cut both lengthways and across, and examine them with a powerful microscope. With the naked eye small points are perceptible, which, magnified, appeared oval or round granulations, at various distances from each other, differing little with regard to size in the same kidney, and which, after a certain degree of maceration, could be detached with the vessels adhering, a void being left in the place which they occupied. They are composed of knotted vessels, surrounded with a grey or ash-coloured substance, and uniting, not so much by anastomosing as by frequently meeting. An injection by the renal artery, if well executed, renders these little bodies entirely red, but in such a manner that some points still appear deeper coloured, and others lighter.

M. Vaus, of Liege, has bestowed much pains in the investigation of the minute structure of the heart, as regards the distribution of the muscular fibres. These are said to be disposed in three layers, of which the outer and middle are common to both ventricles, and the third divided into two parts corresponding to either cavity. The outer layer consists of fine bundles of fibres, which become more and more oblique as they descend, the anterior ones running from right to left, the posterior from left to right; they are continued from the base to the apex, where they intermingle with the middle layer. The fibres of the middle layer follow the same general direction, but are still more oblique, and much more numerous. Some fibres only are said to run so far as the apex, and the others, becoming more superficial, untwist themselves to form the lower layer, which in the right ventricle runs at the back, on the outside, and then before the ventricle, which it at length envelopes on the inside. The lower layer of the left ventricle is much thicker than that of the other; the bundles run from behind forwards, and form the side of the septum; and then, running from right to left, and from below upwards, surround both ventricles, and at length terminate at the origin of the aorta, and the auriculo ventricular opening, extending to the upper part of the posterior furrow.*

### ANATOMY (COMPARATIVE.)

The mechanism of the spine in birds has been particularly described by Mr. H. EARLE, in a paper in the Philosophical Transactions. It appears, from his description, that the cervical vertebræ in this class of animals differ from each other in the form and direction of their articulating surfaces, as well as in the number and shape of the different processes. The articulation seems to be effected by means of joints bearing some resemblance to that of the elbow, but admitting of lateral motion, as well as flexion and extension. Besides this, there is an interarticular cartilage enclosed between duplicatures of the sinovial membrane, analogous to the articulation of the lower jaw in the human species. But, perhaps, the most interesting and peculiar part of the structure is the canal: this is of unequal calibre, being narrowest in the middle, and widening towards the extremities, so that "the canal of each individual vertebra may not unaptly be compared to an hour-glass." The power of motion is further extended by the canal being very imperfect in front, from the existence of a large lozenge-shaped aperture, which is covered with membrane and the ligamentum nuchæ. If the spinal marrow be exposed, by removing the membranes and ligament, "the individual vertebra may be bent backward to a right angle, and laterally to an angle of 45°, without in the least compressing the marrow, which occupies so small a space of the whole calibre of the canal at each articulation," as to be quite secure from any injury from this motion.†

In prosecuting his inquiries on the lymphatic system, M. MAGENDIE dissected a great number of birds and reptiles, by which means he was led to the discovery of some parts of their anatomy hitherto overlooked. An account of these is given in his Journal for April 1822.‡ Some of these organs are situated in the neck, and others in the chest; their form, dimensions, and structure are extremely various, according to the class, order, and even genus. All the birds which he examined presented on either side of the neck, near the trachea, a glandular apparatus, which extends, in general, from the lower jaw and the lower

* *Journal Universel des Sciences Medicales*, Août.

† *Philosophical Transactions*, 1822. ‡ *Journal de Physiologie*, Avril.

part of the head down to the thorax. In the common fowl, and various others, this apparatus assumes the form of distinct isolated bodies, of different size and shape, sometimes contiguous, and at others separated to some distance from each other. In birds of prey, it generally forms a continuous mass from the jaw to the chest,—sometimes extending into its cavity. The colour of these bodies varies, the most common being red, but they are sometimes grey or yellow; their consistence approaches to that of the salivary glands; their paranchema is homogeneous, and completely *sui generis*, no animal structure affording any analogy. The dimensions of these organs vary with the age: in general, they are scarcely to be seen in very young birds, and entirely disappear in advanced life. They receive blood-vessels in abundance, but no nerves have been traced entering them; they have no excretory duct, nor any apparent communication with the neighbouring parts, being isolated in the midst of fat and cellular texture. All the birds, without exception, had two organs in the cavity of the thorax, of an oval or irregular spheroid shape, of a reddish colour, and considerable size, composed in some of two or three distinct parts. Various reptiles presented appearances in some degree analogous; while others, as the lizard, seem entirely to want these organs. M. Magendie seems disposed to regard this apparatus as bearing an analogy with the thyroid and thymus glands: their relative situation, it is true, is the same; but in other respects the resemblance scarcely holds good. The thymus gland is large in young animals, and gradually diminishes; but the converse is the case with regard to the parts described by M. Majendie.

The existence of ducts communicating between the uterus and ovaries in the cow, sow, and some other animals, has been demonstrated by M. Gartner, of Copenhagen. A more detailed account of his observations, accompanied with drawings, is promised.*

The minute anatomy of the nervous system in fishes has been the object of investigation by M. Desmoulins, and given rise to an interesting memoir, the first part of which is contained in the Journal de Physiologie for April; the continuation being given in the Number for October. These observations consist in the detail of facts which do not admit of condensation, and which are too minute to interest the general reader; we must, therefore, content ourselves with having mentioned where they are to be found.

### ANATOMY (MORBID).

Two cases of children born without brains have been recorded by M. Breschet;† and, as the history of such organic deviations throws considerable light on some obscure points of anatomy and physiology, we shall relate them. The first occurred in a male child, which was supposed to be about ten days old, as the umbilical cord had dropped off, and the navel was cicatrized. It lived two days at the Hospice des Enfans Trouvés, being affected with laborious breathing, and in a state of great lassitude. The form and development of the head was perfect;

---

* *Edinburgh Med. and Surg. Journal*, July.
† *Journal de Physiologie*, Août.

the bones were moveable at their sutures; but there was nothing which distinctly indicated the state of the parts within. The cranium and membranes being opened, about twelve or fifteen ounces of limpid fluid, like distilled water, made their escape: this was contained in the cavity of the arachnoid. The dura mater was natural; but the pia mater and the arachnoid membrane were thickened, and very vascular. The brain proper, and its peduncles, were wanting; and there was only a substance, about eight or ten lines in length and breadth, soft, and of a greyish colour, situated anterior to the annular protuberance: this last, as well as the medulla oblongata and medulla spinalis, were natural; but the right lobe of the cerebellum was only half as large as the left. The olfactory nerves were distinctly seen, and two white filaments stretching backwards, but neither the origin nor termination of these could be traced. The optic nerves were of their natural size between the ball of the eye and the cranium, but afterwards became diminished; their commissure did not seem to consist in decussation of their fibres, but to be formed by a small transverse band of union; from this point they diverged, and terminated at the anterior part of the tuber. The third, fourth, fifth, and sixth pairs were likewise traced; and, lastly, no particular appearance was presented, either by the other nerves issuing from the head, or by those of the spinal marrow.

The second case occurred to M. BECLARD. The head was little larger than natural, and was filled with water of a citrine colour, and somewhat viscid; there were cerebral peduncles terminated by eminences, which had the appearance of the optic thalami and corpora striata. The child lived four days.

Although many analogous cases are on record, yet the number is not so great as to take from the interest of those recorded by M. Breschet, particularly as they lead to some interesting considerations on the nature of these anomalies. They would appear to show that congenital hydrocephalus does not depend upon the gradual effusion of fluid causing, by its pressure, the absorption of a brain already formed; but that the growth and development of the brain is first arrested, and the fluid then poured out, by the vessels destined for the nourishment and increase of the medullary substance. If this opinion be correct, it would tend to render the operation of drawing off the water by puncture in such cases even more doubtful than it has hitherto been regarded.

These cases likewise favour the idea, that the brain itself does not give rise to any nerve. That such was the case in those children, is obvious,—all the nerves being perfect, although the brain proper did not exist.

Schirrus of the brain has occasionally been described, but is certainly among the rarest of the derangements of structure which take place in that organ; so much, indeed, that the WENZELS mention only two instances of it as having fallen under their observation. On this account we deem the case related by M. ANDRAL sufficiently remarkable to deserve notice.* The body was opened twenty-six hours after death. Some watery effusion, but not to any considerable extent, was poured

* *Journal de Physiologie*, Avril 1822.

out beneath the arachnoid. The convolutions, viewed externally, seemed somewhat flattened; but, on being cut in thin slices down to the corpus callosum, they presented nothing unusual. On the outer edge of the optic thalamus and corpus striatum of the right side, a body was observed, about four fingers' breadth in length, and two or three in thickness; the surface being rugose, uneven, and of a reddish grey colour. On endeavouring to cut this part with the knife, a resistance was experienced in some points, similar to that which occurs in schirrous affections of the stomach or liver. In these points, accordingly, was found a tissue of a bluish white, semi-transparent, very hard, having here and there small cavities filled with fluid. In short, the change of structure resembled very much, in the opinion of M. Andral, the cancerous masses sometimes found in the liver. The cerebral substance around was healthy. The patient was above fifty; he had suffered, more or less, for fifteen years; the first attack consisting in violent pain, which affected the right temple, spread over the same side of the head and face, and lasted for six weeks. Two distinct periods were perceptible in the history of the symptoms: the first was marked by pain in the head, which at first came on at intervals of considerable duration; these, however, gradually diminished, so that the pain at length became constant. In the second stage, coma, stertor, palsy, and other symptoms of apoplexy, were present. M. BAYLE, who has described this disease, has remarked that the sufferings of those affected are generally prolonged, and that stertor is sometimes present for several days; a fact which was observed in the case before us.

To the numerous observations on the changes of structure which take place in the encephalon, published within the last few years, some of considerable interest have lately been made by M. PINEL, jun. on induration of the brain and spinal marrow, occurring in idiots.* When attentively examined, the indurated portion appears a compact inorganic mass; the colour, consistence, and density of which, strikingly resemble those of a hard-boiled egg. The cerebral substance is sunk and compressed, and seems entirely destitute of blood-vessels. Exposed to the action of heat, it shrivels up, emitting a strong permanent odour, and leaving a dark, compact, shining residuum: whereas, a portion of sound brain, under similar circumstances, expands, gives out but little odour, and leaves a light residuum of a brownish colour. These changes are confined to the medullary matter; at least, M. Pinel has never witnessed them in the cineritious. The nervous substance of the morbid portions tears off in fibres and layers, the direction of which varies in the brain, cerebellum, and spinal cord; affording, in such subjects, an easy means of demonstrating their distribution, according to the manner of GALL and others; and similar, so far as we can judge from M. Pinel's description, to the dissections practised before them by VIEUSSENS and REIL, who indurated the parts artificially by steeping them in alcohol.

Effusions have been described as taking place in the various natural cavities of the brain, and in situations formed by accident or disease;

---

* *Journal de Physiologie,* Août.

but we believe M. VINGTRENIER, of Rouen, to be the first who has detailed the history of such an effusion occurring in the *septum lucidum.** The patient was a female, who died hydrocephalic, idiotic, and epileptic, at the age of twenty; having laboured under these symptoms about fourteen years. Much water was contained under the arachnoid; but, on dissecting down to the lateral ventricles, and opening them, very little fluid escaped, although they had appeared much distended. More minute inspection showed that the water was contained in a cavity, apparently formed by the separation of the laminæ of the septum lucidum; and this to so great an extent as to contain nearly five ounces. This observation would seem to show that the laminæ of this portion of the brain are lined with the same membrane which pervades the other parts, forming a closed sac, and being essentially a serous texture.

A case of tumors in the skull, brain, and dura mater, has been described by Mr. WISHART, of Edinburgh, which is remarkable for the tendency evinced by all these parts to assume this morbid alteration of structure.† The calvarium was of irregular thickness, and in many places even diaphonous; but it was dense, and not easily cut with the saw. The periosteum near the tumor was thickened, indurated, and firmly attached to the integuments. The origin of the tumor seems to have been in the diploe; for the external table was much more eroded than the internal, the latter being only perforated by a small opening. The brain was considerably diseased beneath the tumor; it was soft, fungous, and dark-coloured. The anterior tumor seemed to have originated in the same manner, the outer table being eroded; but the internal, though partly absorbed, was without perforation. The tables of the skull were separated to some extent; and to this circumstance was owing a perceptible swelling, corresponding to the anterior tumor. The space formed between the tables was filled with a white substance, having some resemblance to medullary matter. The pericranium surrounding the tumor was natural, as was the general texture of the dura mater; but on its internal surface were observed a number of tumors, varying in size and form, and being of a fibrous and spongy structure, and easily separated from the parts to which they were attached; depressions in the substance of the brain corresponding to each of them. There was an indurated tumor, the size of a small egg, on the outer and anterior part of the right hemisphere, which seemed to be confined to the cineritious substance, and adhered firmly to the dura mater. When the brain was removed, and the origin of the nerves was examined, corresponding marks of disease were observed; the fifth and seventh being principally affected. A tumor, about the size of a split pea, was attached to the fifth pair of the left side, just at the point of its division; and on the right side were a number of tubercles, about the size of pins' heads. The seventh presented similar appearances; as did the nervi accessorii. Mr. Wishart conjectures that these tumors arose from the dura mater covering the nerves, and not from the proper structure of the nerves themselves. In the cerebellum was a tumor as

* *Revue Medicale,* Juillet 1822.

† *Edinburgh Med. and Surg. Journal,* July.

large as a walnut attached to the upper part of the septum; and the tendency to this formation of tumors *seemed* to extend down the medulla spinalis.

It has been remarked by Dr. BAILLIE, and other eminent pathologists, that the firmness with which the dura mater adheres to the inner table of the calvarium may be so much increased as to constitute disease. Dr. CRAIGIE, of Edinburgh,* is inclined to think that this is not the case, but that, on the contrary, the effect of disease most frequently is to loosen the natural adhesions; a remarkable instance of which he details. A man, about thirty-five years of age, received a slight blow on the head; soon after which he began to complain of pain all over the forehead, and in the course of a few months was affected with amaurosis of both eyes. He next became epileptic, and, after lingering for six months, fell into a comatose state, and died a few days after. The body was examined by Dr. Duncan, jun. and Dr. Craigie. On opening the head, it was found that the scull-cap did not adhere with the usual firmness, "but instantly dropt off, as soon as it was divided all round by the saw." It was unusually thin, and this attenuation was produced by the absorption of part of the structure of the internal table. In some few points, however, the bone preserved its natural thickness, "so as to form the spiculæ of which pathographical writers have spoken, in describing the appearances of the epileptic cranium." These spiculæ, it is to be observed, must have been produced in this case, not by the fresh deposition of osseous matter, but by its irregular absorption from the parts contiguous. The usual arterial connexiont between the dura mater and internal surface of the skull were necessarily absent, and no drops of blood appeared on either. The lateral and inferior parts preseeted the same appearance; and the membrane adhered very loosely to those portions of the basis of the cranium where it is in general attached with great firmness.

Various examples of rupture of either ventricle of the heart are to be found scattered through periodical Journals, or mentioned in pathological essays, almost all of which have occurred in persons advanced in life; and, so far as we can judge, from the cases published by M. ROSTAN,† it would appear to happen most frequently in females. It is also worthy of remark, that the left ventricle appears to give way in the majority of instances, and, in five cases related by the author just mentioned, this took place at the anterior part near the apex. One of the most remarkable cases of laceration of the heart hitherto recorded is to be found in the Number of this Journal for last month. It is detailed by Dr. ASHBURNER, and we must refer our readers to his account for the particulars: at present, we can only mention that both ventricles were ruptured, and in the left several apertures were found. This case, although very uncommon, is not, as its ingenious detailer supposes, without precedent: indeed, one very analogous has been lately published by Dr. BLAUD.‡ This patient was only fifty-eight years of age,

* *Edinburgh Med. and Surg. Journal*, October 1822.

† *Nouveau Journal de Médecine*, Avril 1820.

‡ *Bibliothéque Medicale*, Juin 1820.

and had enjoyed good health until a week before death, when he became affected with pain about the heart, accompanied with a sense of constriction and anxiety. On examination after death, the right ventricle was found ruptured at the anterior surface to the extent of an inch; a similar rupture was discovered in the left ventricle. Besides this, there were three lacerations which did not penetrate the parietes of the heart; two on the surface of the left, and one on that of the right ventricle. In such cases, the structure of the heart seems to undergo a considerable degeneration, becoming thin, particularly at the apex, according to M. Rostan; and so soft and flaccid as to be easily torn. In Dr. Ashburner's case, it is described as resembling wetted brown paper. The observations of Messrs. Blaud and Rostan were published prior to the period embraced in this essay, but we have referred to them in illustration of the interesting case contained in our last Number.

We have nothing to lay before our readers with regard to the lungs, except a well-marked case of melanosis affecting these organs; for which we refer to our Report of prevailing Diseases, contained in the Number of this Journal for December.

Not long ago, a case occurred to a practitioner at Walworth, in which a child was born without an œsophagus. The parts were preserved, and are now in the possession of Sir ASTLEY COOPER. An instance, in all respects analogous, took place in Germany in 1820. The child was born at the full time, appeared healthy, and took the breast as usual; but each time it attempted to swallow, the milk was returned through the mouth and nostrils, in such a manner as to threaten suffocation. It survived, however, for a week, during which time alvine and urinary evacuations took place, although not in the natural quantities. On examination after death, the stomach was found to have no cardiac orifice, but was united to the diaphragm by intervening cellular tissue; the gullet terminated abruptly in a cul de sac, and the œsophagus was entirely wanting. "These cases are interesting chiefly on account of the regular appearance of alvine evacuations which occurred in them both. This circumstance furnishes a very conclusive proof that, at least, the chief part of the intestinal excretions consists of secretions from the mucous surface of the digestive tube, especially from the follicular apparatus, and of the excrementitious parts of the hepatic and pancreatic secretions."*

Many cases are on record in which the stomach has attained dimensions so much above its natural size, as to be mistaken for dropsy of the abdomen; in such instances, the dilatation has, for the most part, originated in obstruction to the exit of the food by the pylorus. But, it is shown by the observations of M. ANDRAL, *fils*,† that it is capable of becoming dilated to an extent almost incredible, independent of this cause. He states that a woman, aged sixty-five, was admitted last March into La Charité, much emaciated, and evidently labouring under organic disease of the stomach; the abdominal parietes had become so much attenuated as to enable M. Andral to trace its dimen-

---

* *London Medical Repository*, August.

† *Journal de Physiologie*, Août 1822.

sions without difficulty. She suffered the usual symptoms of indigestion, and vomited, during nearly forty-eight hours, a large quantity of brownish fluid, which was rejected apparently without effort, and by a process resembling simple regurgitation. The tongue was natural, and the evacuations by stool unfrequent; the pulse very weak, and the skin cold and dry. At the end of about a fortnight she sunk from debility, and the body was examined twenty-two hours after death. The stomach occupied almost the whole of the abdomen: it first descended vertically from the epigastrium, very nearly to the left iliac fossa; then stretched obliquely from left to right, the great curvature being behind the pubis, and resting on the uterus; from the right iliac fossa it ascended to the hypochondrium of the same side, terminating in the duodenum, which, as well as the rest of the alimentary canal, was healthy and undilated. The stomach contained an enormous quantity of brownish fluid; and, for the space of four fingers' breadth next the pylorus, the mucous membrane was wanting, and an ulcerated surface formed; the parts being four or five times their natural thickness, and exhibiting a dense white structure when cut with the knife. The opening at the pylorus was large enough to admit easily the point of the fore-finger.—A similar case is related by the same writer, in which the stomach had attained dimensions nearly as great; and a third is given by M. MAJENDIE, which occurred to him ten years ago, in the person of a relation of the celebrated Talma. This person was seventy-two years old, and, during twelve or fifteen days, received into the stomach a large quantity of liquids, and even aliments, by which the belly became so much distended as to resemble an enormous dropsy. He was then seized with vomiting, by which he evacuated, in a few minutes, as much as filled two large pails. After being thus emptied, he began to fill himself again.—In a case of this kind related by BONNET, the disease was mistaken for ascites, and the patient tapped, by which means the contents of the stomach were evacuated, and the patient died. Here also the pyloric opening was free.

An example of the extent to which the bowels may become distended, is to be found in the third volume of the Dublin Hospital Reports, in Dr. CHEYNE's interesting paper on Dysentery.* The size of the belly gave the patient the appearance of being in an advanced stage of pregnancy. On dissection, the small intestines were found to be seven, and the large intestines nine, inches in circumference.

Two instances of great distention of the kidney have been placed on record: one, in the Edinburgh Medical and Surgical Journal for October, and the other in the Medico-Chirurgical Review for December; the latter being reprinted from the Number of the same Journal for July 1816. In the former, the cortical and medullary parts of the kidney had almost wholly disappeared, leaving nothing but a sac, which consisted of the proper external membrane of the kidney, and the internal membrane much thickened. "It was divided into three large irregular cells, freely communicating with the dilated pelvis, into the apex of which the ureter, of its natural size, opened." About three

* *Dublin Hospital Reports*, vol. iii. and *London Medical and Physical Journal*, November 1822.

pints of fluid escaped, which had the appearance of pus diluted with serum,—not having the smell, taste, or other properties of urine. A calculus was found, exactly corresponding in size to the calibre of the ureter. The tumor was about a foot long, and nine inches wide; it lay behind the peritoneum, filling the whole of the lumbar and part of the iliac region.—In Dr. JOHNSON's case, the patient was seen by various practitioners, "and the general opinion prevailed that the disease was ovarian dropsy." The cyst is calculated to have held five or six quarts of fluid. In both these cases, the inner membrane was highly vascular, and probably secreted the contents of the bag. "It appears, in fact," says Dr. Johnson, "that, from some cause or other, the pelvis of the kidney had become distended to the size of the inner surface of the cyst, while the glandular substance had either become entirely absorbed, or was expanded between the pelvis and capsular tissues, forming, probably, the capillary bodies above described, and still retaining the secretory function."*

Dr. HAVILAND, Regius Professor of Physic at Cambridge, has related a case of what he considers to have been solution of the stomach by the gastric juice after death.† A young man, who had previously been in good health, was attacked with fever, and died. The stomach was removed from the body twelve hours after death: there were two holes in it,—the larger situated on the posterior surface, near the cardiac extremity of the small curvature; it was about half an inch in breadth, and exceeded an inch in length: the smaller was near the other, and about the size of a sixpence. The edges of these perforations were smooth, and well defined. The mucous membrane was more vascular than natural throughout its whole extent, and there were small spots of what appeared to be extravasated blood lying beneath its surface in various places; in others, the coats of the stomach appeared very thin; and in one spot nothing was left but the peritoneum, the other tunics being destroyed. There was likewise a perforation through the muscular part of the diaphragm, large enough to admit the point of the finger: there was no appearance of ulceration, and Dr. H. attributes this likewise to the solvent power of the gastric fluid.

M. NACQUART has placed on record an instance in which the gall-bladder was wanting, having been removed by the progress of disease. A gentleman had an abscess of the liver, which burst externally, and discharged a great number of biliary calculi: after which he suffered, for many years, from hepatic complaints; and, at length, he died, at the age of sixty-eight, apparently of disease of the lungs,—or, at least, his death was much hastened by this occurrence. The liver was reduced to one-half of its ordinary dimensions, but is said to have been in other respects healthy. No gall-bladder was to be found, but the hollow marking its place was filled with a white fibrous substance, bearing some resemblance to cartilage. The cystic duct terminated at the middle of this depression.‡

---

* *Medico-Chirurgical Review*, December 1822.
† *Cambridge Philosophical Society Transactions*, vol. i. part ii.
‡ *Journal Universel des Sciences Medicales*, Août 1822.

M. VALENTIN states, in his Medical Travels in Italy,* that he saw at Naples a gall-bladder, which had two ducts besides the cystic, both separately entering the duodenum.

An instructive case of alvine concretions is to be found in the volume of the Transactions of the Royal Society lately published.† A lad, named Chambers, was in the habit, during the summer of 1814, of eating large quantities of unripe plums, generally swallowing the stones. He pursued his usual occupations till the February following, when he became affected with diarrhœa, and he gradually sunk till the 6th of May, when he died. A tumor (supposed to be an alvine concretion,) had been distinctly felt before death, and, upon opening the body, three concretions were found compacted together in the arch of the colon at the left side, and one lower down near the rectum. This last was cut through, and contained a plum-stone in the centre. Another case is likewise mentioned, in which eight concretions were extracted from the rectum of a boy near Blackburn, two of them about the size of a hen's egg: they are of a light-brown colour, the external coat smooth, consisting chiefly of phosphate of lime and the ammoniaco-magnesian phosphate. Vegetable fibres exist in these concretions, which are supposed to have derived their origin from the oatmeal which formed a large portion of the diet in the case first related.

### PHYSIOLOGY.

Our readers are aware that MAJENDIE has been led to the belief that absorption is not a vital action, but effected by capillary attraction; and that the coats of all blood-vessels, as well arterial as venous, possess a physical property by which we may explain all the phenomena of absorption. These conclusions were deduced, among other premises, from the result of certain experiments, which consisted chiefly in carefully detaching portions of great vessels,—separating them from the surrounding parts by the introduction of a card,—and applying poison (generally the nux vomica,) to the coat of the vessel. Under these circumstances effects were produced, varying in duration and intensity, according to the thickness of the vein or artery. Similar experiments have been performed by Dr. HUBBARD, of Philadelphia,‡ but with results entirely different: in the third of these only did the poison appear to affect the animal, and then the effect is attributed, with apparent probability, to its absorption by other textures. "Here we might have been led into a gross mistake, by attributing to its being absorbed through the veins effects which were undoubtedly owing to the operation of the poison in contact with other parts. But, upon examining the card, which was very thin, and, together with the vein, was kept constantly moistened, we found its under surface tasted strongly of the poison, the card being saturated with it. To this circumstance, undoubtedly, are attributable the slight effects produced." After this occurrence, a piece of sheet-lead was substituted for the card, and no

---

* *Voyage Medicale en Italie.* Par le Docteur LOUIS VALENTIN.

† *Philosophical Transactions for the year* 1822, part i.

‡ *Philadelphia Journal,* August 1822.

effect resulted from the application of the poison. From these experiments, Dr. Hubbard concludes "that the large vessels do not possess in their coats a physical property—capillary attraction, by which fluids are carried directly into their cavities;" while, with regard to the small vessels, he objects, that the only experiments of the French physiologist were performed on dead matter. But it appears to us, that by far the most striking experiment of Majendie has been passed by Dr. Hubbard without any attempt at refutation; it is this:—a dog was rendered insensible by opium, and the thigh separated from the body in such a manner as to leave it attached by nothing but the crural artery and vein, the cellular coat being very carefully removed. Two grains of upas were now thrust into the paw, and the effect was as sudden and intense as if the limb had not been separated at all. Again, in a second experiment, the barrel of a quill was introduced into the crural artery, and another into the crural vein; they were fixed in the vessels with ligatures, between which the coats of the vein and artery were divided. The poison was introduced in a similar manner, and with similar result.

Our object in briefly referring to these very curious experiments, is to afford an opportunity of introducing the explanation of them given by Dr. Good, in his recent work.* It was the opinion of Cruikshank, and probably of the Hunters and Hewson likewise, that lymphatic absorbents entered into the coats of veins and arteries very freely; and, therefore, Dr. Good thinks there is no difficulty in supposing that the poison might enter the lymphatics, and thus *accompany* the veins. An obvious difficulty occurs, however, in admitting this explanation in those experiments where the coats of the artery and vein were both divided: but here, too, the learned author has an ingenious, although to us not quite satisfactory, answer. He observes, that the lymphatics anastomose more frequently than any other set of vessels, and that their valves are sometimes placed at a considerable distance from each other, occasionally not less than six inches. These valves alone prevent the possibility of the contents regurgitating; and hence, in M. Majendie's experiments, it is conjectured that the poison was first taken up by the lymphatics, and afterwards stimulated to a retrograde motion; and thus, ultimately, thrown into the stream of blood by the mouths of those lymphatics that enter the coats of the veins. Besides, it is remarked that in the dead body the valves lose their elasticity so as to allow fluids to pass in any direction; and hence it is inferred that, in the state of pain and debility produced by these experiments, the parts may, in the same manner, lose their power of resistance.

So entirely different, however, are the conclusions of Dr. Hubbard from those of M. Majendie, that he does not suppose the nux vomica, prussic acid, and similar poisons, are absorbed at all, or pass into the circulation either directly through the sides of the blood-vessels, or by the medium of lymphatics; but that they produce their effects upon the nerves alone. And, further, that it is necessary, in order to secure the success of the experiment, that the nerve be left *in situ*. Where a card

---

* *Study of Medicine*, vol. ii. p. 279.

was insinuated beneath it, the poison was applied without injury; but, when this was not done, the effects were in proportion to the importance and extent of the nerves acted upon, and apparently affected by no other circumstance, "except, as was in some cases suspected, by the division of small blood-vessels."

Drs. LAWRANCE and COATES* have likewise been engaged in a course of experiments on the subject of absorption, which have led to some interesting results, tending to illustrate the chemical changes which may take place in substances contained in living vessels, into which they have been introduced by the process of absorption. A solution of the sulphate of iron was thrown into the abdomen of a cat, and a solution of prussiate of potass into the cellular texture of the abdominal parietes, taking care that no portion of the solutions should be mixed. The fluid in the thoracic duct became of a deep blue; the coagulating lymph of the blood and the lungs were likewise strongly tinged with the same colour, and the urine more slightly, although quite distinctly. The shortest period required in kittens for the prussiate to arrive at the upper part of the duct, varied from two to five minutes. It is to be observed, that none of the salt was ever found in the blood or other fluids, until it had made its appearance at the upper part of the thoracic duct;—a fact militating against the doctrines of Majendie. That the contents of absorbent vessels continue their course for a certain period after death, has frequently been supposed; but there does not seem to be any satisfactory proof of additional matters entering them from without:—in the *lacteals*, indeed, an appearance of this kind is described by Majendie. With a view of determining this question respecting lymphatics, Drs. Lawrence and Coates bled kittens to death, and then injected prussiate of potass into the abdomen; and, in one instance, tied all the larger vessels at the heart, so as to prevent any circulation. In every experiment, "absorption of the foreign matter took place distinctly, as evinced by the usual test applied to the fluid of the thoracic duct, but in considerably longer time than during life."

In our Number for June will be found an account of some interesting Experiments on Absorption, by a Committee of the Academy of Medicine of Philadelphia.†

The experiments of Majendie do not, however, rest entirely upon his own testimony: they have recently been repeated, and ingeniously varied, by M. SEGALAN.‡ One of the most remarkable and most favourable to Majendie's views of absorption, is that in which he isolated a portion of intestine, cutting off all connexion between it and the rest of the body, except by means of an artery and vein, and then injecting poison into the bowels: the death of the animal so treated was the result. An objection may be made to this experiment,—viz. that the absorption of the poison used might arise from the peritoneum, in consequence of a certain portion of it remaining on the part of the gut

---

* *Philadelphia Journal*, August 1822.
† *Ibid.* No. 6.
‡ *Journal de Physiologie*, Avril 1822.

exterior to the ligature. To answer this, M. Segalas repeated the process, but did not return the intestine into the abdomen; and still the same effect followed, although more slowly. He likewise attempted to prove the venous absorption by negative results. A portion of intestine was isolated by two incisions, and the blood-vessels tied, taking care to exclude the lacteals, rendered visible by the absorption of chyle. The same poison (viz. an aqueous solution of alcoholic extract of nux vomica,) was thrown into the bowel, and retained there; yet no effect was produced. To this experiment it may be objected, that the circulation being interrupted, the power of absorption might be lost from the want of this usual stimulus; and, consequently, that this example does not apply to the natural state of the part. To meet this objection, the experiment was repeated, leaving only one large artery to supply the intestine with blood: the same negative result followed. But it may be said that the want of venous circulation may have caused a congestion, as unfavourable to absorption as in the preceding case. In the next experiment, therefore, the vein corresponding to the artery was left untied, but detached from the mesentery in such a manner as to give exit to the blood; yet the poison did not act. Lastly, after leaving a dog, in the foregoing experiments, under the influence of the nux vomica for an hour without effect, the natural circulation was restored by taking the ligature off a vein, and the animal was poisoned in ten minutes. These experiments, performed again and again at the Hospice de la Faculté de Medecine, and before a certain number of pupils, have always been attended with the same results.

Considerable attention has likewise been paid to the state of the absorbent system by M. ANDRAL, which, although attended with negative results, seem worthy of being recorded. Cruikshank, Mascagni, Soemmering, and Saunders assert that they have found bile in the lymphatic vessels of the liver;—Mascagni, that he has seen the thoracic duct, and the lymphatics of the thorax and abdomen, filled with a red fluid resembling blood;—Soemmering, that he has detected calcareous particles in the absorbents leading from carious vertebræ;—and Dupuytren, in a case of abscess in the thigh, found pus in the lymphatics of the groin. M. Andral, on the contrary, has frequently examined the bodies of persons dying under circumstances similar to the above, but never found similar appearances. In a few instances, the fluid contained in the thoracic duct has been of a reddish colour,—such as is observed in animals that have fasted long; but this appearance never presented itself in cases of sanguineous effusions, to the re-absorption of which Mascagni attributed the phenomenon. Neither has he ever detected bile in the lymphatics of the liver. This evidence, therefore, is corroborative of that of Majendie, who says that, although he has often examined the lymphatic system, he has never found any thing there that could reasonably be suspected to have been absorbed.

Perhaps the experiments of Dr. FOHMAN, of Heidelbergh,* may tend, in some measure, to reconcile these jarring and contradictory statements. We allude to the discovery that there exists in the

* *Salzburgh Mediiinisch-Chirurgische Zeitung*, No. 46.

intestines of the lion, and some other animals, a direct communication between the lymphatics and veins, which was demonstrated by injecting the former with mercury, which flowed into the latter. As this anastomosis has subsequently been found in the dog, cow, horse, and some species of phoca, it seems not improbable that more careful investigation may lead to its detection in man likewise. An indirect communication of this nature has been demonstrated by Professors TIEDMANN and GMELIN: they injected the absorbents of the intestines in the dog, horse, cow, and human body, with mercury; the metal in every instance found its way into the mesenteric veins and vena porta. But, as they contained the mercury immediately on their exit from the mesenteric glands, and not till then, it appears reasonable to conclude that the communication took place in these bodies.*

The assertions of various experimentalists, that mercury was not to be detected in the secretions of those whose systems were nevertheless under its action, have led several to deny its absorption: among others, a very assiduous physiologist, Mr. SWAN, who has lately attributed its effects to the medium of the nerves.† We pointed out in our last Number the negative nature of the evidence on which this and similar hypotheses are founded: some evidence in favour of the opinion we then advanced has since come under our notice, which we deem of sufficient importance to record. In HUFELAND's Journal, for November 1820, is an account of a skull found in a burial-ground, the osseous texture of which was penetrated with quicksilver; and in the same Number of the Journal it is mentioned that, in the Cabinet of Midwifery at Lubben, there is the pelvis of a woman who died of syphilis, in which the diploe are infiltered with mercury. But an instance of a nature still less equivocal has recently been published by M. DUBLANC, of Paris.‡ M. B. accidentally swallowed a gold ring, which alarmed him so much that he applied to M. Dublanc, who quieted his apprehensions by assuring him that it would be evacuated by stool; a prognostic which was fulfilled next day, and M. B. returned, not a little pleased at having got rid of his unwished-for riches, but still more astonished at its appearance,—the ring being, as he thought, transmuted from gold to silver. The application of heat restored the natural colour, and the phenomenon was satisfactorily accounted for by the circumstance of M. B. being under a mercurial course by means of *frictions.* It is worthy of remark, that these had been discontinued for a week, showing that mercury may still remain in the system a considerable time after its further introduction has been suspended.

The effects of deleterious effluvia have been investigated by Dr. J. C. ROUSSEAU, with results which, if confirmed by future experiment, must be regarded as new and important.§ He had long suspected that the palate and mouth act but a very secondary part in the discrimina-

* *Versuche über die Wege auf welchen Substanzen aus dem Magen und Darmcanale ins Blut gelangen, über die Verrichtung der Milz und die geheimen Harwege.* Von H. TIEDMANN und L. GMELIN.

† *An Inquiry into the Action of Mercury on the living Body*, by JOSEPH SWAN, &c.

‡ *Bibliothèque Medicale*, Août 1822.

§ *Philadelphia Journal*, No. vii.

tion of taste; indeed, are only sensible to the mechanical action of the substances applied to them; and that the most nauseous or the most grateful, the most inodorous or the most aromatic substances, act alike upon the palate, while it remains unassisted; but are readily discriminated, even in their minuter shades of variation, as soon as the breath loaded with their effluvia is transmitted to the nostrils. The sensation he regards as being always proportioned to the rapidity with which the column of air passes through the nostrils, and thinks it may be entirely destroyed by stopping them with sufficient accuracy. In illustration of this position, various experiments are detailed; two of which were performed on medical gentlemen, who were sceptical as to the accuracy of Dr. R. "A professional gentleman, with whom I had several arguments on the present subject, unwilling to concede that the sensual pleasure derived from eating and drinking rested entirely and exclusively in the olfactory organs, consented to trust his conviction to experiments. He was, for this purpose, blindfolded, and his nostrils were rendered impervious by compressing them between the fingers. In this state, a small lump of camphor was put in his mouth, and masticated for some time: unable to determine the nature of the substance, acting then solely upon his tongue and palate, he declared it to be a piece of crumb of bread with pepper on it." Another professional friend, still more sceptical than the preceding, next became the subject of experiment. "Having previously been blindfolded, with his nostrils well secured, a small portion of assafœtida was substituted for the camphor used in the preceding experiment, and put into his mouth. He turned and turned it again between his tongue and palate, and exultingly exclaimed that it was camphor. His mortification cannot be described in finding himself so severely disappointed, when he was restored to the exercise of the senses he had momentarily been deprived of." The experiments were varied by inhaling the effluvia of various volatile oils, but it was only by passing the breath through the nostrils that these were distinguished from each other; and similar trials were made with regard to tobacco, by plunging individuals into baths made of the infusion, when no effect was produced until the nostrils were opened, even although they were allowed to remain in, an hour and a half or two hours. No degree of intoxication was produced by breathing the vapour of spirits, so long as the nostrils were secured; but the subject of the experiment became so much affected when the vapour passed through his nose, as to stagger at the end of half an hour. Dr. Rousseau thinks that his observations, if correct, would seem to point out the Schneiderean membrane as the weak and vulnerable part by which the influence of contagion is received, and that miasmata, existing under a form capable of being carried by the atmosphere, are thus constantly applied to the inner surface of the nostrils.

There is, perhaps, no object in physiology of greater interest than M. MAJENDIE's experiments upon the functions of the roots of the spinal nerves. Mr. CHARLES BELL had pointed out to the notice of physiologists, that nerves having single roots seem also to have single functions; while nerves having a double origin have also double functions. The fact, however, seems never to have been brought to the test of experiment in a manner so satisfactory as by Majendie,* who appears not to have been entirely aware of the advancement made by Mr. Bell in the same path of discovery, as he states that he "had not entertained the slightest idea of what would be the probable result of this experiment." He exposed the posterior half of the spinal marrow in a puppy, and, by proceeding carefully, was enabled to expose the posterior roots of the lumbar and sacral nerves; these were subsequently divided with a pair of fine scissars, without injuring the spinal cord. The limb, when pricked or violently pinched, was, to all appearance, without any sensibility or power of motion; when, to his surprise, the animal began to move it very distinctly. A second and third experiment was followed by similar results; and M. Majendie, being assured that the sensibility was destroyed while the power of motion remained, was led to suppose that the posterior roots gave sensation without motion. To prove the converse of this hypothesis, it became necessary to divide the anterior roots, leaving the posterior untouched. This, however, was an experiment more easily planned than executed. After various unsuccessful attempts, M. Majendie found that, by tearing off the dura mater from the spinal marrow, he could display the anterior roots united in bundles, just where they pierce that membrane,—and so divide them. The limb was altogether deprived of the power of motion, yet its sensibility was retained. These experiments were repeated and varied on different animals, and the results, both with respect to the anterior and posterior roots, were confirmed in the most satisfactory manner. Such is the first paper of M. Majendie; a second has been published in the Number of his Journal for October, the principal part of which we subjoin.

He then wished to ascertain whether it were possible to divide the anterior or posterior roots of the spinal nerves, without opening the covering of the spinal marrow; for, on exposing this to the air and cold, the nervous power is sensibly diminished. The anatomical disposition of the parts did not render this impossible, for each fasciculus travels for some way in a canal proper to itself before it joins the other. In effect, he found that, with the aid of a blunt-pointed scissars, he could remove the parts sufficiently to lay bare the ganglion of each pair of lumbar nerves; and then the canal which contains the posterior roots could be separated by a small stylet, without much difficulty, and subsequently divided. This manner of conducting the experiment afforded the same results as had previously been observed; but, as the experiment is much more difficult, M. Majendie prefers the method described in his first paper. Being desirous to ascertain whether convulsions would be excited in a member, the nerves of which had been divided, and if they would take place to the same extent as usual when the nerves of sensation were destroyed, he applied the nux vomica, with the following results:—In an animal in which the posterior roots were cut, the muscular contraction was as great as if the origin of the nerves had remained untouched: on the contrary, in an animal in which the nerves of motion were divided, the member which they supplied

---

* *Journal de Physiologie,* Août.

remained supple and unaffected at the time when the other muscles of the body were powerfully contracted by the influence of the poison. In pinching or pricking the posterior roots, the animal showed symptoms of pain, but not nearly to the same extent as when the spinal marrow was touched, although slightly, at the place where the roots take their rise. Almost every time that the posterior roots were thus irritated, contractions were excited in the muscles supplied by them: these contractions, however, were infinitely weaker than if the spinal marrow itself were touched. The same trials were made with regard to the anterior roots, and, as might be expected, with opposite results; the contraction being powerful, and even convulsive, while scarcely any symptom of sensation was evinced.

The parts were next subjected to the action of galvanism: in the first instance, leaving them in their natural state; and, in the second, dividing the spinal extremity, so as to place it on an isolated body. Contractions were produced by means of either root, but those excited by the anterior were in general much stronger, and more complete, than when the posterior were the media of the electric influence.

M. Majendie informs us that he is now occupied in tracing the phenomena of sensation and of motion in the spinal marrow itself; and it is but justice to this industrious experimentalist to state, that he gives the merit of originality to Mr. Charles Bell, limiting his own claims to the performance of the ingenious experiments which we have above detailed.*

Within the period allotted to this Essay, a continuation of Mr. Bell's first paper, in the Transactions of the Royal Society, has been published: it relates to the nerves which associate the muscles of the chest, in the actions of breathing, speaking, and expression. He informs us that "The nerves on which the associated actions of respiration depend, and which have been proved to belong to this system by direct experiment and the induction from anatomy, arise very nearly together. Their origins are not in a bundle, or fasciculus, but in a line or series, and form a distinct column of the spinal marrow. Behind the corpus olivare, and anterior to that process which descends from the cerebellum, the corpus restiforme, a convex strip of medullary matter, may be observed; and this convexity, or fasciculus, or virga, may be traced down the spinal marrow, betwixt the sulci, which give rise to the anterior and posterior roots of the spinal nerves.

"This portion of medullary matter is narrow above where the pons Varollii overhangs it. It expands as it descends; opposite to the lower part of the corpus olivare it has reached its utmost convexity, after which it contracts a little, and is continued down the lateral part of the spinal marrow.

"From this track of medullary matter on the side of the medulla oblongata arise in succession, from above downwards, the portio dura of the seventh nerve; the glosso-pharyngeus nerve; the nerve of the par vagum; the nervus ad par vagum accessorius; the phrenic, and the external respiratory nerves.

---

* *Journal de Physiologie*, Août et Octobre.

"It is probable that the branches of the intercostal and lumbar nerves, which influence the intercostal muscles and the muscles of the abdomen in the act of respiration, are derived from the continuation of the same cord or slip of medullary matter. Nor will it escape observation, that the nerves called phrenic and external respiratory, though coming out with the cervical nerves, do, in all probability, take their origin from the same portion of the medulla spinalis with the accessory nerve.

"The intercostal nerves, by their relations with the medulla oblongata, are equal to the performance of respiration, as it regards the office of the lungs; but they are not adequate to those additional functions which are in a manner imposed upon the respiratory apparatus, when they are brought to combine in other offices."

In the usual state of the body, Mr. Bell regards the intercostal muscles as sufficient for the performance of respiration; but, under circumstances which render the breathing more difficult, the diaphragm, the trapezius, the serratus magnus, and the mastoid muscles, are called into action, and it is to these alone that the nerves called *respiratory of the chest* are distributed.

"If we examine the par vagum, the portio dura of the face, the external thoracic, the diaphragmatic, and the spinal accessory nerves, by comparative anatomy, we shall conclude that they are all respiratory nerves, by their accommodating themselves to the form and play of the organs of respiration. In fishes, the respiratory nerve goes out from the back part of the medulla oblongata. When it escapes from the skull, it becomes remarkably enlarged, and then disperses its branches to the bronchiæ and the stomach. But from the same nerve go off branches to the muscles moving the gills and operculum, whilst a division of the nerve is prolonged under the lateral line of the body to the tail. It is said this division sends off no branches, but this is not correct; it gives branches in regular succession to the muscles from the shoulder to the tail. Experiments have been made upon these nerves, but their detail would lead us too far. It is scarcely necessary to add, that there is neither phrenic nor spinal accessory, nor external thoracic nerves, in fishes,—the order of their muscular system not requiring them. In birds, the structure of the wing, and the absence of the mastoid muscle, render the spinal accessory nerve unnecessary; it is wanting for the reason that, in the absence of the diaphragm, there is no phrenic nerve. Quadrupeds have the three respiratory nerves of the trunk; but even in them there are variations in the muscular frame, which illustrate the appropriation of the nerves. The construction of the neck of the camel is like that of birds: there is a succession of short muscles along the side of the neck, and attached to the vertebræ; but there is no long muscle, like the sterno-cleido-mastoideus, contributing to the motion of respiration. There is, accordingly, no spinal accessory nerve in the neck of this animal.

"We have a remarkable example of the manner in which these nerves vary in their course of distribution, and yet retain their appropriate functions, in the nerves of the neck of birds. In them, the bill precludes the necessity of the portio dura going forward to the nostrils and lips; the nerve turns backwards, and is given to the neck and throat; and it is particularly worthy of remark, that the action of raising the feathers of the neck, as when the game-cock is facing his opponent, is taken away by the division of this nerve."*

These nerves are likewise regarded as organs of expression, and their functions further illustrated from pathology; but our limits will not admit of our entering more minutely into the subject. For an excellent general view of the opinions and experiments of Messrs. Bell and Shaw, we refer our readers to the paper, by the latter gentleman, inserted in our last Number; and to the Medical Intelligencer, No. 36, for a clear and impartial analysis of all that has been published on this subject, with the exception of Mr. Wallace's paper,† and that of Mr. Shaw just alluded to. A full account of Mr. Mayo's experiments and remarks was given in this Journal for November.

### PATHOLOGY.

Some observations occur in the two last Numbers of the Edinburgh Medical and Surgical Journal on the subject of Purpura Hemorrhagica; and, from the dissection of two cases, it appears evident that the petechiæ are not confined to the dermoid system. Indeed, this fact had been ascertained before, and an interesting case is to be found in the Number of this Journal for April, in which appearances presented themselves similar to those about to be described. In both the above cases, purple spots were observed studded over the viscera of the thorax and abdomen; and these were not confined to the serous textures, as the internal surface of the heart, stomach, and bladder, were spotted in one or other of them. In Dr. DUNCAN's case,‡ "the integuments were, perhaps, more livid than usual; the tongue was much thickened; there was gangrene of the velum pendulum palati, the tonsils, and pillars of the fauces; the glottis and epiglottis were inflamed; but the redness did not extend into the larynx. The pleura was marked with petechiæ, and there was an ecchymosis on the right side of the spine; the lungs were healthy; the heart was beautifully studded with petechiæ, some florid, some deep red, some purple; the colon, meso-colon, and mucous membrane of the stomach and of the bladder, presented the same appearance." It is remarkable that in none of the three dissections alluded to was the brain examined. Dr. Duncan very justly calls in question the propriety of ranging this among the cutaneous diseases,—not only because the hemorrhage is much more important than the spots on the skin, but because dissection shows that they are not confined to this part, but are likewise found internally. He regards the disease as essentially a hemorrhage, for each petechia is, in fact, a little ecchymosis; and the great peculiarity of the affection is, that small bleedings take place at very distant parts of the body, and from different textures. The modes in which he regards it as possible for this disease to arise are,—"1. Increased tenuity of the blood, allowing it to escape from the superficial extremities of the minute arteries. 2. Dilatation of the mouths of these arteries, allowing natural blood to

* *Transactions of the Royal Society*, 1822.

† *Quarterly Journal of Foreign Medicine.*

‡ *Edinburgh Med. and Surg. Journal*, July.

escape. 3. Tenderness of the coats of the minute vessels giving way, from the ordinary impetus of the blood. 4. Increased impetus of the blood, rupturing healthy vessels. 5. Obstruction in the vessels, causing rupture, with natural impetus, and without increased tenderness. 6. Two or more of these causes may act simultaneously, or successively." It is difficult, as Dr. Duncan remarks, to conceive any other way in which purpura can arise; and yet none of these explanations are easily reconciled to the phenomena of the disease: for it is very questionable whether increased action of the vascular system does generally precede the eruption, and still more difficult to believe that tenderness or softening of the minute vessels should take place simultaneously all over the body. He thinks it more easy to suppose that a sudden relaxation of the mouths of the vessels may take place at the same time, or that the disease proceeds from tenuity of the blood, which, when it has arrived at a certain pitch, escapes through the open mouths of all the vessels of a certain size.

These cases have given rise to some judicious remarks from Dr. WHITLOCK NICHOLL,* who observes, that, from Dr. Duncan's analysis of the modes in which this affection may occur, it is evident that venesection is warranted only by the fourth cause which he has assigned for the origin of purpura, namely, "increased impetus of the blood," except in those cases where the "obstruction in the vessels" arises from an overloaded state of the circulating system. Should this state be referable to obstruction existing in the viscera, as of the spleen or liver, he conceives purgatives will be particularly useful. There remain the first, second, and third heads of Dr. Duncan's analysis, and for these Dr. Nicholl proposes the oleum terebinthinæ; two cases successfully treated by this remedy having been published by him in the London Medical Repository for July 1821; and, on the present occasion, a third is added. A languid, pallid child, two years and a half old, was brought to him a few days after she had become covered with purple spots all over the skin and in the mouth. A drachm and a half of the oil of turpentine was given daily, in divided doses, with syrup of senna and water; under which treatment the eruption gradually disappeared between the 1st and 11th of December; and, returning in March again, yielded to a similar mixture, *alternated with bark and mineral acid.*

Some very interesting and important remarks on Partial Paralysis, from the pen of Mr. SHAW, are to be found in the last volume of the Medico-Chirurgical Transactions.† The object of these is to show that the most common cause of such affections of the face is not in the brain, as has often been supposed, but that it generally depends upon some injury or disease of the portio dura, called by him "the respiratory nerve of the face." The first series of cases relates to the effects of inflammation of this nerve, and, among others, the state of a person named Richardson is described. On looking at him, there does not appear

---

* *Edinburgh Med. and Surg. Journal,* October 1822.

† We did not analyze Mr. SHAW's paper along with the others in the Medico-Chirurgical Transactions, intending to give a general view of the new doctrine regarding the functions of the nerves called "respiratory." The paper by Mr. S. in our last Number has rendered this, for the present, unnecessary.

any thing unusual about his face, but the moment he smiles it is drawn to one side. When he laughs or sneezes, the distortion is very remarkable. In sneezing, particularly, the muscles of the nose, mouth, and even eyelids, of the right side, were passive, at the time all the muscles of the other side were acting strongly. The right buccinator did not correspond in action with the left, so that, when he attempted to whistle, the air escaped by the right angle of the mouth; but the patient could bring the orbicularis oris into action, and could hold a pencil with it; and he could shut his jaws with equal force on either side. The sensibility of the paralyzed cheek was also equal to that of the other. In short, all those actions which have been proved to depend on the influence of the fifth pair of nerves remained perfect. This, it will be at once observed, is just the reverse of what generally holds good with regard to paralysis following apoplexy, in which those actions enumerated as perfect in the foregoing example are deficient, and those connected with the portio dura are little, if at all, affected. In Richardson's case, the previous history agrees well with the views of Mr. Shaw; for he had suffered a very severe attack of cynanche parotidea, having his face so much distorted as to lead the people, in whose house he lodged, to the belief that he was mad.

Several cases of a similar nature are detailed, and the various inconveniences arising from this cause are illustrated: among these, the most distressing seems to be the loss of power in the eyelids of the paralyzed side: the eye being thus deprived of its natural covering, and constantly exposed to the light, becomes destroyed by repeated attacks of inflammation. This consequence is particularly striking in the case of a porter named Garrity, as described by Mr. Shaw. About nineteen years ago, he was affected with violent pain in the right ear, accompanied with discharge: the face became paralyzed on the same side; since which time he has never been able to close the right eyelid, in consequence of which he has suffered repeated attacks of inflammation; the cornea has become opaque, and the sight is nearly destroyed. Another remarkable appearance presented by this patient is the extreme thinness of the cheek, owing to the muscles having been so long unemployed; while the masseter and temporalis muscles of the same side are not at all diminished in size. "During the course of last winter, a surgeon in town was much distressed by receiving intelligence that one of his relations in the country was attacked with symptoms of paralysis of the face; but, in the course of a short time, he was not less surprised than pleased to hear that his friend had been suddenly relieved of all the symptoms by the bursting of an abscess in his ear. The progress of this case puzzled my friend not a little, until he read the account of the inquiries in which we had been engaged in Windmill-street."

The views of the ingenious author of this paper are not confined to those cases of partial paralysis in which some local pressure, or other injury, affects the portio dura, but he proceeds to illustrate the connexion between this form of paralysis and that depending on mischief within the head. A woman was attacked with pain in the right ear, accompanied with discharge, and paralysis of the side of the face: the discharge stopped, and she was seized with pain in the head, vertigo, and some degree of stupor. From these she recovered by active treatment.—A

child was affected with discharge from the ear: some time after this had ceased, she was seized with a fit, and fell down; there was total paralysis of the face, leg, and arm of the same side. These cases show the propriety of examining the state of the ear in partial paralysis of the face, accompanied with pain or other unpleasant symptoms about the head.

Another cause of this local palsy seems to be exposure to cold, commonly called *a blight*. Such instances are said to be common in India during the prevalence of cold winds; and Mr. Shaw informs us, that it is sometimes to be observed "in those unfortunate females who walk the streets during the night." In such cases it, for the most part, gradually subsides; while the palsy accompanied with inflammation and suppuration is extremely difficult of cure. In order to attempt this, however, Mr. Shaw suggests leeches and blistering,—it would appear from analogy, as he gives no example of his having employed it himself.

Injuries of the head likewise give rise occasionally to partial paralysis of this kind, one of the most interesting examples of which is thus related. "While accompanying M. Cloquet, one of the most ingenious and learned surgeons in Paris, in his morning-visit to the Hospital St. Louis, he drew my attention to the case of a woman who had some unusual symptoms, produced apparently by a fracture of the clavicle. On examining her, I discovered all the appearances of paralysis of the portio dura; and, on further inquiry, I found that, at the time the clavicle was broken, she had received a blow on the temporal bone of the same side."

Exactly analogous is the effect of dividing the nerve in operations; and it becomes a question of great importance to surgeons, whether greater inconvenience may not result from such division, than from allowing any tumor of moderate size to remain. The restoration of continuity following the division of other nerves, and the return of power, is sufficiently ascertained; but more extensive observations and experiments are required to settle this question with regard to the portio dura. The evidence at present is rather against its restoration.

M. Serres has given an account of several cases of inflammation, or apoplexy, of the cerebellum,* attended with symptoms so peculiar as to lead to the detection of the seat of disease even before death.—The first of these occurred in 1814. A man, aged thirty-two years, was brought to the Hôtel Dieu in a state of insensibility, with his face red, his head hot, his pulse strong, his breathing slow and interrupted, with occasional convulsions and tonic spasms; and with the penis in a state of permanent erection, which remained until within four hours of his death. He died on the twelfth day. The hemispheres of the brain presented no mark of disease, but the cerebellum was much injected with blood; the superior vermicular process, and the processus cerebelli ad testes, being of a deep red colour, and containing several small *foyers*. The opinions of Drs. Gall and Spurzheim regarding a connexion between the genital organs and cerebellum, naturally suggested

* *Journal de Physiologie*, Août 1822.

itself, and directed the author to further observation, and several analogous cases, of subsequent occurrence, are related. Among these, one of the most remarkable is that of Marie-Jeanne-Josephine Dubourg, who died at the age of thirty-three, in consequence of habitual and excessive indulgence in venery. Connexion with the other sex not satiating her desires, she became addicted to masturbation, which was followed by stupor, and at length, tired of habitual gratification, she submitted to have the clitoris burnt. This operation failed to deaden her passions, and she returned to the same method of satisfying them, till at the age of thirty-two, she suffered from violent head-aches, and became suddenly imbecile. The vermiform processes were indurated, and the surrounding part of the cerebellum inflamed ; but its general texture was softened, resembling that of a fœtus of the second month ; the arteries of the cerebellum were unusually large. The body had been injected, with a view to ascertain the state of the vascular system, and the uterine, vaginal, vesical, and hemorrhoidal arteries were found very much dilated.

A case of a similar description, and appearing to coincide with the views of the French pathologist, is given by Mr. DUNGLISON, in the London Medical Repository for October. A boy, aged five years, was attacked with increased action in the head, evinced by convulsions, strabismus, coma, &c. with convulsive motions of the right side, and palsy of the left: these symptoms proved fatal at the end of eleven days. The penis had been observed, at different times, in a state of complete erection, and diseased appearances were found, by dissection, in the cerebellum and its membranes.

The great extent to which the brain may sometimes be injured, without producing immediate death or any mental derangement, is well known to pathologists, and especially familiar to military surgeons. The fact is well illustrated by a case which recently occurred in Paris, and which is recorded by Dr. DUPONCHEL.* A dragoon was wounded in a duel, the sword of his opponent entering the skull through the orbit, and beneath the ball of the eye. The degree of injury could not, of course, be ascertained at the time; but, on dissection, the sabre was found to have divided the optic nerve and the ophthalmic artery, and passed completely through the substance of the middle lobe of the right side, penetrating even to the calvarium, but leaving the ventricle untouched. A portion of cerebral matter was found in the orbit, having been brought there in forcibly withdrawing the instrument by which the wound was inflicted. Slight effusion, with some partial adhesions, were found about the right hemisphere, but there was no appearance of suppuration in any part of the brain. The patient lived sixteen days ; the symptoms being paralysis of the left side of the body, dilated pupil, disposition to coma, and difficulty of speech, but with the mental faculties unimpaired till a few hours before death.

M. VALENTIN, in the work already alluded to, mentions having seen at Rome a preparation of the brain, in which a hair-pin was found, about two inches long, which had been thrust perpendicularly into the

* *Bulletin de la Société d'Emulation de Paris*, Mai 1822.

fontanelle, and passed down between the hemispheres as far as the corpus callosum, which, however, it did not touch. It was situated on the left side of the falx, and had not wounded the longitudinal sinus. The head of the pin was in contact with the dura mater, but had left no mark on the bone. This singular circumstance is related of a Russian soldier, who lived to the age of thirty-six, and had served in the Roman army: he died of inflammation of the lungs, and was opened by S. FLAJANI. As this plan of pushing pins into the head is said to be common in Russia as a means of infanticide, it is conjectured that the foreign body had remained in this instance from the period of very early infancy. If this be correct, it is probable that the pin must, when introduced, have transfixed the corpus callosum, as it is stated that, when the head was examined after its full development, it reached down to that body.

It is singular that, in the various treatises upon Diseases of the Eye, we hear so little of the Lachrymal Gland: connected as it is with our mental and bodily sympathies, we should, *a priori*, have expected it to be particularly subject to morbid actions, but this does not seem to be the case. BOYER remarks, that the maladies of this gland are little known, because its situation both protects it from external injury, and prevents our being able to examine it during life. He states that he has never known an instance of its being inflamed; nor does he seem to have been aware of any other instance of disease, except that an instance of scirrhous enlargement of the gland, in which extirpation was performed, had been presented to the Royal Academy of Surgery in France. Mr. TODD,* however, informs us that he has met with many instances of disease of this organ, and that such affections are therefore less rare than occulists have been inclined to suppose.

The author first treats of *acute inflammation* of the lachrymal gland, which he states to be sometimes idiopathic, but more frequently succeeding to some form of ophthalmia. He has likewise known it to accompany the psorophthalmia of children, when it was severe or aggravated by adventitious circumstances. The symptoms which he regards as characteristic of this disease are intense pain "under the temporal extremity of the eyebrow," extending to the neighbouring parts, sometimes into the cranium, with profuse or defective secretion of tears: when the latter is the case, the palpebræ and cheek become excoriated to some extent; both eyelids, but particularly the upper, are tense and swollen; and, lastly, the inflammation, spreading to the conjunctiva, gives rise to a severe and obstinate form of ophthalmia. "When inflammation of this kind is at its height, there are much symptomatic fever and restlessness; flushing of the face, particularly at the affected side; acute pains darting through the orbit and head; and, as it is stated (by Waller), more or less delirium, with strabismus, and impaired vision and protrusion of the globe, proportionate to the size of the swelling."

*Chronic inflammation* of the lachrymal gland is regarded as, for the most part, of strumous origin; it is marked by an obvious enlargement

* *On Diseases of the Lachrymal Gland*, by C. TODD, &c. &c.—*Dublin Hospital Reports*, vol. iii.

at the outer part of the orbit, frequently causing an œdematous swelling of the upper eyelid, and difficulty in moving the eye towards that side; and is to be remedied by leeches, blisters, and the usual means employed in struma. Scrofulous enlargement is an affection of the lachrymal gland, which Mr. Todd regards as well marked; it is distinguished by the tumor presenting a surface "more or less lobulated," by the absence of pain, and the slowness of its progress; sometimes continuing stationary for many months, and in others running on to strumous suppuration.

*Abscess* is said frequently to succeed to acute inflammation, occasioning much pain and tension. Mr. TRAVERS, in his Synopsis of the Diseases of the Eye, mentions suppuration of the lachrymal gland as a frequent occurrence in children, and recommends evacuating the matter from the inner surface of the eyelid,—probably in order to avoid disfiguring the parts by leaving a cicatrix. To this, however, Mr. Todd objects, that deformity seldom succeeds, unless from delaying the operation till the integuments become diseased; and therefore recommends that incisions should early be made parallel to the superior margin of the orbit, by which means few fibres of the orbicularis will be cut, and the cicatrix, "being in the direction of the natural folds of the eyelid, will be concealed by them."

*Schirrus* of the lachrymal gland has chiefly been regarded as accompanying a general cancerous or carcinomatous state of the eye; but the author shows, by a case given at length, that it may exist as a distinct disease. The gland was extirpated: it was much larger than a walnut; presented considerable eminences, with fissures between them; and was nearly as hard, but more elastic, than cartilage. On being cut into, several small cysts were discovered, containing a glairy fluid, the interstices consisting of fatty substance, with transverse membranous bands. Much coagulated blood filled the cavity after the operation, and gave to the eye the appearance of being in a gangrenous state. The patient, however, did well; and, "although vision was *totally extinct* in that eye, yet the orbit was *perfectly sound*, and its position almost natural." Another case is given, in which Mr. O'BEIRNE extirpated this gland, although without discovering what the tumor was, till it was laid bare by the incision: vision remained unimpaired, and the patient suffered "no inconvenience whatsoever from the loss of the gland." This operation has likewise been successfully performed by Mr. Warner and Mr. Travers.

A disease of the Cellular Membrane of the Throat, which would seem to be of rare occurrence, has been described by Dr. GEORGE GREGORY.* The patient complained of pain and tenderness of the external parts of the throat, extending round the neck, and being particularly felt at the junction of the clavicles with the sternum. She had considerable fever, with much difficulty of swallowing, but no diseased appearance could be perceived on examining the fauces. There was neither hoarseness nor any considerable difficulty of breathing. She was bled and purged, without any effect in arresting the progress of the disease, which, conti-

---

* *London Med. and Phys. Journal,* October.

nuing to advance, proved fatal on the seventh day. The cellular membrane surrounding the trachea, pharynx, and palate, was every where in a state of disease: "in some places, actual sphacelus had occurred; in others it was in a state of what might be called imperfect suppuration." The same morbid alteration extended along the anterior mediastinum, as low as the ensiform cartilage. The thoracic and abdominal viscera were sound; and the mucous membrane of the throat, larynx, and pharynx, presented no other unusual appearance, except that they were covered with an abundant secretion of tenacious mucus. This form of disease, to which Dr. Gregory proposes to give the name of Cynanche cellularis, is regarded by a respected contemporary as a modification of erysipelas. "The above disease (says he,) appears to us to be an unequivocal specimen of that dangerous species of erysipelas which attacks the cellular membrane under the skin, and between the muscles. In the year 1809, the crew of his Majesty's ship *Royal Oak* suffered severely from this disease, while cruising in the Bay of Biscay. Some lives were lost, and the disease appeared to be contagious, running through a considerable number of the ship's company. It would destroy the whole cellular membrane of a limb in a few days, and, when the integuments were slit open, they would fall completely off the muscles, which were left as clean as if they had been carefully dissected. Nothing but free and early incisions through the integuments, so as to allow of the exit of matter and cellular sloughs, saved the life or limb. In several instances it attacked the trunk of the body, and two or three cases proved fatal. The carpenter, Mr. Dalrymple, died in a state of coma. The fever attending the disease was of a typhoid type, and did not bear depletion."*

In our Number for September, we mentioned a case, described by Dr. O'Brien, in which the larynx was ruptured by external violence. Emphysema took place, not only of the external parts, but in the anterior mediastinum; and the blood in the heart, aorta, and vena cava abdominalis, likewise contained air. Dr. Duncan, jun. regards the production of the subcutaneous emphysema as easy of explanation; but not so the *pneumothorax,* which he supposes to have been the immediate cause of death. The left lung in this case was very much diseased, and could contribute very little to the function of respiration; and, therefore, when the right lung became collapsed and compressed by the entrance of the air between the pleura costalis and pulmonalis, suffocation necessarily followed. On laying open the chest, the right lung was collapsed; and it was ascertained that this state had existed prior to the opening of the cavity, because the air was felt by Dr. O'Brien to rush out, when he made the first incision through the cartilages of that side. Dr. Duncan says, the first idea which suggested itself was, that the lung had been ruptured by the external violence; but this was disproved by the fact of its being easily inflated; and he thinks the only rational supposition is, that air, blown into the cellular membrane of the neck from the ruptured trachea, was at last forced

* *Medico-Chirurgical Review,* December 1822.

into the cavity of the chest through some fissure in the pleura costalis, although none was discovered.

Some interesting observations on Emphysema, and its consequences, are to be found in a paper by Mr. SHAW.* He was sent for to examine the body of a patient, who had died in a state of great emaciation from disease of the larynx, whose body became blown up with air, and quite putrid, within forty-eight hours after death. This phenomenon Mr. Shaw supposes to have originated in the difficult and obstructed state of the respiration, in a manner similar to the emphysema which sometimes occurs in women during labour; the putrifaction having been hastened by the respired air lodging in the cellular membrane. The reasons assigned for supposing the respired air to act on dead animal matter, (that it does not affect the living body, is proved by every day's experience,) are, that he has several times witnessed this phenomenon,—particularly in the bodies of two very powerful young men, brought to the dissecting-room in Windmill-street soon after they had been drowned. They became black, and puffed up about the neck and chest, within twenty-four hours, although it was in winter; and, as a dead body does not become emphysematous in consequence of lying in water, till after a considerable time,—"indeed, not for weeks, during the winter,"—Mr. Shaw supposes that a certain quantity of air must have escaped through a rupture in the bronchia or trachea, during the convulsive attempts to breathe. Proceeding upon the idea that the injection of the cellular texture with inspired air had facilitated putrifaction, and still continued to do so, portions of the skin were removed so as to allow its escape, and the gas pressed out: the parts thus freed from the air which had been respired, did not run into putrifaction so rapidly as those which remained under its influence, although they were now of necessity exposed to the action of the atmosphere.

A full and authentic statement of the case of Cummings, the American sailor, who swallowed, at different times, thirty-five clasp knives, has been given by Dr. MARCET; but the circumstances are so well known, that we deem it unnecessary to recapitulate them here. Certainly not the least interesting part of Dr. M.'s communication, is the account drawn up by Cummings of his adventures, and which was found after his death.†

A similar instance to that of Cummings is detailed in a recent American Journal.‡ A young man, who is asserted to have been long in the habit of swallowing various indigestible substances, as musket-bullets, billiard-balls, buttons, &c., about the beginning of June of the present year, swallowed fourteen knives in one day,—he gradually sunk, and died on the 25th of August. Two of the knives had been passed; one was found in the œsophagus, and the rest in the stomach. This individual is said on one occasion to have swallowed a gold watch, with the chain and seal; they were evacuated on the ninth day, darkened in colour, but not otherwise altered.

In a recent Number (for November,) we described, in our review of

---

* *London Med. and Phys. Journal*, September.
† *Medico-Chirurgical Transactions*, vol. xii. part i.
‡ *New-York Medical Repository*, October 1822.

M. CRUVEILHIER's late work, the peculiar pulpy softening of the mucous membrane of the stomach and bowels, which sometimes takes place in children.* Similar appearances, occurring as a result of inflammatory action in various parts, have been found by M. LALLEMAND, the general position of this pathologist is, that softening of the organic structures is one of the most general effects of acute inflammation.†

A singular variety of Urine has been described by Dr. MARCET, in which the fluid, although colourless when voided, became nearly black soon after.‡ Several cases of a similar kind have since been mentioned.§ In one of these the urine was of so deep a colour, that the brightest object, put into the vessel containing it, could not be perceived; and there was no sediment, although it had stood many hours. The exact relation of this phenomenon with the general health is not sufficiently ascertained, although it does not seem distinctly indicative of disease, Dr. Marcet's first patient, and the one just alluded to, having been quite well. Another example, mentioned by this writer, occurred in a girl subject to daily attacks of a febrile and hysterical nature: in Dr. COPLAND's case, there was "an inactive state of the liver;" and in the one mentioned by Dr. MACLEOD, the patient was affected with pain across the region of the kidneys, and considerable general debility. The chemical peculiarity of this urine seems to be that it contains neither lithic acid nor urea, but that it owes its black colour to a compound of a peculiar principle with ammonia. According to Dr. PROUT,|| this new principle seems to be one possessed of acid properties, although, from the small quantity of the specimen which could be had for his experiments, it was impossible to obtain satisfactory evidence of its nature. Should future investigations confirm these conjectures, it is proposed to distinguish this new acid by the name of *melanic*, from its black colour.

Various phenomena have attracted notice of late years, which seem to show a connexion between the diseases of the human race and the lower animals, much greater than was at one time supposed to exist. Hydrophobia, cow-pox, and the diseases of horses called glanders, exhibit proofs of this; and another illustration will be found in a subsequent part of this Essay, in which a short account is given of a disease affecting cows and other animals in some parts of America, and communicated by them to individuals using their milk or eating their flesh. The only instance, however, of any of the lower animals being affected with Croup, we believe, is contained in an observation by M. DUPUY, professor at the Veterinary School of Alfort, read before the Royal Academy of Medicine in August last.¶ The dog was about three or four months old, and was brought from a neighbouring village, in which

---

* *Médecine Pratique, eclairée par l'Anatomie.* Par M. CRUVEILHIER.

† *Recherches Anatomico-Pathologiques sur l'Encephale et ses Dependances,* par F. LALLEMAND; and *Journal Universel des Sciences Medicales,* Juillet.

‡ *Medico-Chirurgical Transactions,* vol. xii. part i.

§ *London Medical Repository,* August and November; and *London Medical and Physical Journal,* August.

|| *Annals of Philosophy,* August.

¶ *Bibliothéque Medicale,* Août 1822.

the croup was prevalent at the time: during his illness, he had the same shrillness of voice which is found in children labouring under this complaint; frequently opening his mouth, as if to expel some foreign substance lodged in the pharynx, and speedily dying under symptoms of suffocation. On opening the body a few hours afterwards, a false membrane was found in the larynx, of a reddish colour, which extended into the divisions of the bronchia; and the lungs were filled with an abundant serous effusion. This observation of M. Dupuy is of the more importance, as the complaint was supposed to be hydrophobia; a mistake extremely likely to occur in dogs dying with symptoms of strangulation.

### SEMIOLOGY.*

Various accounts have been published in America† of a fatal disease, supposed to be produced by taking into the stomach the milk or flesh of animals, particularly cows, which is supposed to have acquired deleterious properties from feeding upon some poisonous plants at Tennessee. This is conjectured to arise from the "Indian hachy," a plant used medicinally by the Cherokee Indians, the systematic name of which the writer (Dr. M'Call) was unacquainted with. So great is the virulence of this poison, that the small quantity of milk usually taken with tea is said to be occasionally followed by fatal consequences. The symptoms are nausea, vomiting, vertigo, confused vision, and fever, with irregular exacerbations. The pulse is variable, being sometimes strong and at others tremulous, while the skin is hot and parched, the eyes red, and all the secretions diminished. The disease, when fatal, terminates in paralysis and coma before death, which takes place about the sixth or seventh day. In one case, a number of flattened pustules were observed, in different states of maturity, the largest of which might have been covered with a shilling, and from which a dark grumous fluid issued on slight pressure. Dogs, cats, hogs, buzzards, crows, or any other animal, which eats of the tainted flesh, is liable to the disease. A description analogous in all the leading characters is given by Dr. Asa Coleman of the disease as it appears in the state of Ohio, except that he mentions great lassitude, with pain in the calves of the legs, as preceding for a few days the symptoms we have enumerated above; and that, about the third or fourth day from the commencement of the vomiting, the patient experiences a slight pain in the head, ending in delirium and phrenitic symptoms. According to the writer last mentioned, the disease attacks both sexes indiscriminately, and all ages, although it is most severe in persons of middle age. The mortality, however, is not so great as might be imagined from the severity of the symptoms, as it does not appear that above one in twenty, or, according to some, one in thirty, die, even of those who experience the complaint in its severest form. With regard to the treatment, emetics are said to be hurtful, and cathartics beneficial,—before administering which it is found necessary to quiet the stomach by a few grains of

* In the introduction, this department was called "Medicine," through inadvertency on the part of the Editors.

† *Philadelphia Journal.—Western Quarterly Reporter.*

carbonate of potass, with or without opium. The sugar of lead is another remedy, said to be of much use in allaying the vomiting; and a large blister over the stomach is likewise enjoined.

A different view of the origin of this disease has been given by Dr. LEA, of Tennessee,* who attributes it to the effects of malaria. Without pretending to give a decided opinion on this question, we may remark, that some of the circumstances mentioned by Drs. M'Call, Coleman, and Haines, who have all written on the subject, militate strongly against Dr. Lea's explanation. Among other facts, it is stated that the disease prevails in some of the western counties of Ohio, on the Miami and Scioto rivers; confined, however, to limited tracts of country, *in which there is no peculiarity as to humidity, dryness, or elevation.* In one instance, a piece of woodland was enclosed as a sugar orchard, in a dry and elevated situation: any animal allowed to graze here became affected, although they remained the whole season without injury in a field of pasture adjoining. It likewise principally attacks those animals that have come from a distance, or have been reared in cultivated ground, by which they are deprived of the opportunity of becoming habituated to the plant, or learning to avoid it. Sucking calves, which use only the milk of the mother, become affected when the milk is thus tainted; and animals allowed to feed on flesh of a similar description show marks of the disease. "I saw (says Dr. Coleman,) an instance of a whole family becoming sick with this disease, some of them a few hours after dining upon a loin of veal, in which it was afterwards satisfactorily ascertained that the calf laboured under the disease at the time it was butchered, being sold at market by an unprincipled person." It would appear that a cow may have the milk secreted in such a state as to prove poisonous to calves, dogs, and other animals, while she herself does not appear to suffer; and hence some of the American writers are disposed to believe that the deleterious principle is peculiarly eliminated and excreted by the latiferous system.

### STATISTICAL MEDICINE.

For an account of the diseases prevailing in London during the last six months, we refer to various Reports under this department in the preceding Numbers.

To hear of Small-pox after Vaccination is unfortunately nothing new, yet we conceive it our duty to notice some additional information on this subject, which has been published during the period embraced in these remarks.† Dr. JOHN FORBES, physician to the Chichester Dispensary, has given an excellent account of a small-pox epidemic, which prevailed in that town and its vicinity last winter and spring. The tract of country which was the seat of his observations consisted of parts of the counties of Sussex and Hampshire, stretching along the coast about twenty miles, containing many populous villages, with the town of Chichester nearly in the centre. There appears to have existed in this district an opportunity particularly favourable for observing the comparative effects in the vaccinated and unvaccinated, because small-

* *Philadelphia Journal,* No. iii. † *London Med. Repository,* Sept.

pox inoculation being little, if at all, practised, the great majority of individuals consisted of those guarded by vaccination, or else wholly unprotected. In June, 1821, the small-pox was introduced by the child of an itinerant player, from whom several individuals caught the disease. The surgeons of Chichester and the neighbouring villages having declined inoculating with small-pox, the epidemic might possibly have speedily subsided, but for the interference of an ignorant and assuming individual, one Pearce, a farmer, supposed to inherit, both from his father and mother, extraordinary skill in inoculating, and even power over the progress and degree of the eruption. To this person the sagacious overseers of the parish of Bosham applied to inoculate the pauper children with small-pox, by which means these gentlemen had soon the satisfaction of seeing the disease spread over the whole district, containing a population of 30,000 souls. Mr. Pearce, thus sanctioned by the authority of such important personages, became in great request, and a knife-grinder, a fishmonger, and a whitesmith, observing the new trade of their neighbour to be abundantly lucrative, set up as rival practitioners, and by under-selling, and other means, in imitation of their betters, contrived to rob the farmer of about one thousand patients, to whom they communicated the disease. The surgeons of the district, finding small-pox raging among the people, (who, by the wisdom of the overseers of Bosham, could by no means avoid exposure to contagion,) were now driven to the necessity of inoculating for small-pox; and the number to whom the disease was thus artificially communicated by the professional and ex-professional inoculators is supposed to have considerably exceeded three thousand. Of those inoculated by the regular surgeons, about 1 in 200 proved fatal; of the others, no accurate account could be obtained. Of 130 or 140 individuals affected with natural small-pox, twenty died.

During this epidemic, the individuals who had been vaccinated were exposed, directly and designedly, to the influence of small-pox, with the effect of showing, in many instances, very remarkable power of resisting its contagion. In one family, the four elder had been vaccinated, and seven were now inoculated with small-pox: all the former escaped infection, though living, and some of them even sleeping, together. About eighty cases, however, did occur, in which persons previously vaccinated became affected with small-pox; but it proved extremely mild in every instance, except one. Six hundred and eighty persons, who had been previously vaccinated, were inoculated with small-pox; and of these about thirty showed constitutional affection, the eruption consisting, for the most part, of a few pustules. Nineteen well-authenticated examples occurred of secondary small-pox: these appear to have been much less modified than small-pox after cow-pox usually is, the attack having been in some instances extremely severe, and in two it had nearly proved fatal. The opinion of the surgeons of the district is, that the disease was not in any degree modified by the preceding attack; but they can only speak positively in this respect of one-half the number above mentioned, the others not having been seen during the latter stages.

An account of considerable interest has likewise been published of the small-pox as it prevailed at the other extremity of the island,*—Caithnesshire; and which, at the same time, forms a striking instance of the rise and progress of a disease truly contagious in its nature. Dr. JAMES INNES, of Wick, was called to a patient whom he found labouring under small-pox; and, the disease not having been prevalent for ten or fifteen years, he was induced to make particular inquiries into its origin. This was easily discovered: the individual in question had arrived a few days before from Leith, at which place the small-pox was then raging epidemically, and he had lodged in a house where several children had the disease. This patient proved a focus from which the contagion spread with inconceivable rapidity, and gave rise to small-pox, which raged over the country for the space of four months. The account of Dr. Innes relates principally to the phenomena of the disease in those who were guarded by previous vaccination,—between four and five hundred persons so guarded having been affected with varioloid eruptions during this epidemic. About eight-ninths of these had the disease in the form known by the name of *horn-pock;* ten only had it severely, constituting coherent small-pox; and, in thirty cases, the eruption appeared to be purely vesicular from its commencement to its termination. Seven cases are mentioned of the recurrence of small-pox: in one, the first eruption was *varicelloid,* and the second well-marked variola of the coherent kind; two had passed through inoculated small-pox, and in them the second eruption is stated to have been as mild as the first; four had laboured under the natural form of small-pox, in three of them the second eruption was milder than the first; in the fourth, an old woman, who had lost the sight of one eye during the first attack, the secondary was more severe than in any of the others alluded to. However mild the forms of eruption proved in those protected either by previous vaccination or by having had the small-pox, yet when we turn to the less fortunate individuals who remained entirely unprotected, no doubt can for a moment exist with regard to the true nature of the disease. Here it exhibited all the formidable varieties of this loathsome complaint, in many instances proving fatal even when it was not confluent; and almost all who were attacked with it in this most aggravated form fell victims; pectoral symptoms of the worst kind supervening, accompanied with hemorrhage from the lungs, hæmaturia, and bloody stools. The phenomena displayed by this disease radiating from one individual, as from a centre, and spreading over an extensive district, afforded admirable opportunities of answering the question,—may all the varied forms of varioloid eruption arise from one and the same contagion? Dr. Innes answers this, without hesitation, in the affirmative; having had occasion to witness every possible form of the disease in persons living under the same roof,—"confluent small-pox in one,—coherent small-pox in a second,—distinct in a third,—the vesico-pustular in a fourth, and the pure vesicular disease in a fifth."

We think our readers will agree with us in regarding the statement of Dr. Innes (whose veracity we hold to be unquestionable, from personal knowledge,) as one of the strongest that has yet been published in favour of the identity of origin of all varioloid eruptions, as held by

* *Edinburgh Med. and Surg. Journal,* July.

Dr. Thomson,* Dr. Macleod,† and others. Small-pox, we are told, "had not prevailed epidemically during the last ten or fifteen years." A person labouring under that disease arrives from Leith; its appearance was so little familiar, that inquiry was instituted to ascertain where he could have come by it; clearly showing that the disease was not known,—or, considering the smallness of Wick, we may rather say, *known not* to exist in the town. "From this young man's case may be dated the origin of the epidemic in this part of the country; and it is inconceivable with what rapidity it spread, attacking a great proportion of the unprotected, and not a few even of the vaccinated." As it spreads, every form of eruption presents itself, from the most malignant to the mildest, and that among individuals living under the same roof. In thirty cases it proves "purely vesicular, from its commencement to its termination;"—that is to say, it is genuine varicella, even according to the new and restricted definition given of that eruption by some of the Edinburgh pathologists. We are free to confess, for our own parts, that we cannot conceive evidence more satisfactory on a point of this nature.

Evidence, of an irrefragable nature, of the general occurrence of small-pox after cow-pox, is to be found in the foreign Journals, particularly in those from America; essays on this subject having been published by Dr. JAMESON, of Baltimore, Dr. MITCHELL, of Philadelphia, and Dr. DAVIS, of Columbia. We cannot, therefore, but express our astonishment and regret that any should continue to deny the truth of these and similar accounts;‡ astonishment at resisting such a body of evidence,—regret, because we are convinced it is not the way to remove the prejudices against vaccination. We must open our eyes, and look the evil in the face; otherwise, how can we learn to counteract it?

The history of small-pox has been ably investigated by Dr. THOMSON, of whose work we would speak more fully, but that it is our intention to review it at length in an ensuing Number.

### THERAPEUTICS.

The numerous and apparently satisfactory cases of Phthisis Pulmonalis, stated, in the work of Sir A. CRIGHTON, to have been cured by the inhalation of Tar-vapour, induced Dr. FORBES, deputy inspector of military hospitals, to give this remedy a trial:§ "for, (as he justly remarks,) however sceptical we may be with regard to the power of any remedies in producing any thing beyond a partial alleviation, or temporary retardation, of this cruel disease, yet it was possible that a remedy had at length been found capable of alleviating the sufferings of the patient, and, by arresting the progress of the symptoms, affording some chance of permanent cure." These trials were made at the General Military Hospital, Fort Pitt, under the direction of Dr. Forbes; and,

---

* *Account of Varioloid Epidemic in Scotland,* by Dr. THOMSON. 1820.

† *On the common Origin of all Varioloid Eruptions,* by Dr. MACLEOD.—*London Med. and Phys. Journal,* July and August, 1820.

‡ See Dr. JENNER's letter to Dr. GREGORY.—*London Med. and Phys. Journal,* September.

§ *London Med. and Phys. Journal,* October.

from the result, he states his belief that the cases recorded by Sir A. Crighton as having been cured by the inhalation of tar-vapour were not genuine phthisis, but more nearly allied to chronic catarrhal affections, between which and tubercular consumption the diagnosis is frequently attended with much difficulty. The statement of Dr. Forbes is given in the plainest and most satisfactory manner, and the experiment seems to have been conducted with the utmost care and attention to every circumstance likely to influence its results; yet in not one case, out of nineteen in which the vapour was tried, did it give "evident proofs of its power either to arrest or to ameliorate the symptoms of the disease." —"In general, it rendered the breathing more difficult, and cough almost incessant; the expectoration was diminished, the pulse commonly accelerated, and anxiety and restlessness produced." The manner in which the remedy seemed to produce its deleterious effect was by diminishing the quantity of the expectoration; and, in proportion as this happened, the cough became more frequent, and the breathing more oppressed. In some, it appeared to cause no other inconvenience than thirst and head-ach; but, even where it did not excite greater disturbance, in no case was it regarded as of the smallest benefit.

Although the inhalation of tar-vapour would appear to be thus inefficient in the tubercular phthisis, Dr. Forbes was led to a more favourable opinion of its power in chronic catarrh; understanding by that term a disease succeeding previous inflammatory action of the bronchiæ, but which does not itself consist in organic lesion or actual inflammation, having for its cause a morbid relaxation of the mucous membrane of the air-passages, which gives rise to an increased secretion. Tar-vapour appears to possess stimulant properties, "and perhaps astringent ones," and, consequently, to be a remedy peculiarly adapted to restore the energy of the parts. Proceeding upon this principle, Dr. F. administered the vapour in this class of complaints; and, of thirty-two who were submitted to its action, eight were cured, six improved; in eighteen it produced no effect, and in none any bad effect.

The results of Dr. Forbes's experiments seem to agree with those of Hufeland and Neumann, who tried it in the Charity Hospital at Berlin,* in fifty-four cases of what is called "consumption;" but, from the circumstance of four having been *cured*, we are inclined to be sceptical as to these last having been genuine phthisis pulmonalis. The point of coincidence, however, is, that, according to the German physicians, the vapour was most beneficial in "blenorrhœa" of the lungs, or "phthisis pituitosa ulcerosa *atonica*," which probably depends on a state of the mucus membrane lining the air-passages analogous to that in "chronic catarrh," as described by Dr. Forbes. In Hufeland's experiments, two wards were appropriated to it, which were kept filled with the vapour by a large pot of tar placed in the middle. Fifty-four cases were submitted to the inhalation; of these, four were cured, and six much relieved; sixteen received neither good nor harm by its use; twelve got worse, and sixteen died.

The treatment of Inflammation of the Respiratory Organs by means of the Tartarized Antimony, has for a considerable time been the

* *Bericht über die zur Prüfung der Werkung der Keerraucherungen bei der Lungensucht, &c.*—Journal der Practischen Heilkunde. Erstes Stück, 1820.

fashion in some parts of the continent, particularly Italy; and, although it has not yet, nor indeed seems likely at any time, to gain ground in this country, we still conceive it to be our duty to lay before our readers the evidence that appears in its support. At Naples, the plan is to administer small doses of tartar emetic in solution, following it up with digitalis and nitre. At Milan, however, RASORI is in the habit of exhibiting the medicine in much larger doses, giving, in some cases, above seventy grains in the course of twenty-four hours.*

In addition to the Italian physicians, M. PESCHIER,† of Geneva, has recently stept forward as an advocate for the treatment of inflammation of the chest with antimony, to the exclusion of blood-letting; and his statements are more decidedly in its favour than those of any other writer. He has used it for five years in the Canton de Vaud, during which time he states pleurisy and peripneumony to have been twice epidemic; and that, while his less fortunate professional brethren lost a considerable proportion of their patients, *he cured all his without any exception.* His plan, when he is consulted in such cases,—whether chronic or recent, slight or severe, with fever or without,—is to administer from six to twelve or fifteen grains in six ounces of water, which is to be given in the course of twenty-four hours, at divided doses; some laxative tisanne being taken every hour. The dose of tartar emetic was generally increased three grains a-day, till the patient took twelve or fifteen; while, at the same time, any indication which nature seemed to hold out was attended to. Thus, if there was a disposition to sweating, two drachms of nitric, muriatic, or acetic æther, were added; if there was much pain, one or two drachms of tincture of opium; if dysuria and dry skin, a like quantity of nitre. The effects were, that the patients generally vomited after the second and third spoonsful; then the medicine acted on the bowels, or perhaps produced no sensible effect, but speedily removed the disease. It was, besides, observed that a large dose of tartar emetic produced less vomiting than a small one; and that, when a grain or a grain and a half only were given in the twenty-four hours, it caused distressing fits of retching, without beneficial result. Although vomiting and purging generally followed its exhibition, yet the antimony is stated to have acted equally well in removing the disease where no sensible effect was produced; the cure being generally completed within eight days, and rarely requiring fifteen. It was never deemed necessary to bleed, either generally or locally; but blisters were sometimes applied over the seat of pain.

Dr. ARNAUD, of Moulins, has likewise published a paper, in which he details numerous cases of pleurisy and peripneumony cured by antimony, without bleeding; and M. LAENNEC communicated to the Academie Royale de Médecine, at Paris, some cases of hydrocephalus and inflammations of the chest similarly treated. M. L. thinks the sequelæ of the inflammation more quickly removed by the tartarized antimony than by blood-letting. The mortality is calculated at one only in forty at Naples, where this system is in repute; while at Rome, where the new doctrine is less generally received, it is said to amount to forty-five in the hundred.‡

---

* *Voyage Medicale en Italie, fait en l'année* 1820. Par LOUIS VALENTIN.

† See *London Medical Repository*, November.

‡ *Voyage Medicale en Italie, &c.*

We may take this opportunity of mentioning that the *nuova dottrina*, as it is called, continues to engage the attention, and to receive the strenuous support, of many of the most eminent men in Italy. The principles and history of this doctrine have been so fully entered into in preceding Numbers of this Journal, that it would be a work of supererogation to recur to them at present.

A curious illustration is afforded of the effects of counter-irritation, in an interesting paper by Mr. OGDEN.* A boy, six years of age, had been affected from infancy with cough, accompanied with copious expectoration; to which epilepsy had been, for two years, superadded: this, however, was apparently connected with the state of the alimentary canal. The child's clothes caught fire, by which it was so severely burnt that the skin was removed from all the fore-part of the chest, and from a considerable part of the abdomen. The remedy, though severe, was effectual,—the cough and epilepsy being entirely removed.

The propriety of exhibiting Emetics in cases of obstinate Constipation has been insisted upon by Dr. HOSACK, of New York, and illustrated by the relation of various cases.† An account of these will be found by referring to our Number for August.

Camphor has been recommended in the cure of some forms of Inflammation, by Dr. BÖDTCHER, of Copenhagen. His plan is to use camphor in the form of vapour, generally as an application to the parts affected; and it is recommended for inflammatory affections of the eyes, nostrils, fauces, and lungs. In ophthalmia, it is to be held before the eye; in cynanche, the vapour of warm water is to be impregnated with it, so as to admit of its inhalation; and the same form is to be employed in coughs, croup, and pneumonia. It is likewise supposed to be of service when rheumatism or gout leave an external part, and attack some internal organ: in such cases, keeping a piece of camphor in the mouth is regarded as the most efficacious manner of using it.‡

Dr. HARDEN, of St. Petersburgh, has proposed a new application of the cold affusion,—viz. to the cure of Croup. "The patient was his own daughter, a child of eighteen months, who had already suffered two attacks of the disease. As all usual remedies, such as leeches, emetics, purgatives, &c. in this last fit were used in vain, and the child seemed to be near her last agony, the unhappy father resolved to try the affusion of cold water. A cushion filled with hay was put into an empty bathing-tub, and the child placed in it, with her belly upon the cushion; upon which Dr. H. seized a pail filled with water of 12° R., and poured it quickly several times, from the head along the spine, but in a manner by which the greatest power was directed on the reverse of the breast. The symptoms, after this first affusion, became a little better; and after, at intervals, the operation had been ten times repeated, the child became better, and recovered. Affusions of cold water were made use of by Dr. Harden, with equal success, in the first

---

* *London Med. and Phys. Journal*, August.

† *Observations on the Use of Emetics in Constipation of the Bowels.* By DAVID HOSACK, M.D. New York.

‡ *Bibliothek for Læger*, a Danish Journal.

inflammatory stage of croup; in proof of which he relates two cases.—Dr. Miller, another physician of St. Petersburg, also cured a case of croup in its last stage with cold affusions, and observed the same effects as Dr. Harden."*

The calamitous death of the late Primate of Ireland has directed the attention of medical men, in an especial manner, during the last few months, to the means of removing poisons from the stomach, or counteracting their effects. In the London Medical Repository for July and August will be found interesting papers on the treatment of those who have swallowed large quantities of opium, by Mr. Wray, Dr. Copland, and Mr. Sprague; and, in the Numbers of our Journal for September and November, papers by Mr. Bush, of Frome, and Mr. Jukes, of Westminster, on the means of evacuating the stomach mechanically where emetics fail to operate. The principal feature of novelty recommended in the treatment is cold affusion. The water is "to be dashed copiously, and with considerable force, upon the head:" by this means, the sensibility of the system appears to be roused, and vomiting may be brought on with more readiness,—a circumstance particularly mentioned in the case related by Dr. Copland. Mr. Sprague keeps the patient in the erect posture, with cloths dipped in cold water to the head, while the lower extremities are to be immersed in water as hot as can be borne. He gives various formulæ for emetics, enemata, &c. and suggests that very beneficial effects arise from the stimulus of a single drop of the liq. volat. cornu. cervi, cautiously insinuated into the external canthus of the eye.

Even if all these methods fail, a recourse is left in the very ingenious contrivance of Mr. Jukes, which consists in evacuating the stomach by introducing a flexible tube, attached to an elastic gum bottle filled with water: the bottle being pressed, its contents are forced through the tube, and distend the stomach so as to prevent any of the poison from lurking in its rugæ; and, the pressure on the sides of the bottle being again relaxed, it assumes its former dimensions, being filled with the contents of the stomach, which rush into it on the principle of the syringe. This is to be continued and repeated until the water thrown into the stomach returns unimpregnated with opium.—Mr. Bush's method consists in using the syringe instead of the bottle, and he suggests the propriety of placing the patient on the left side. Although neither of these gentlemen are strictly entitled to the merit of originality, as a similar plan is described by Orfila,† yet the claims of both are better founded than those of many of our pretenders to improvements in medical science. The experiments of Mr. Jukes, in particular, are of unparalleled boldness, and show the full confidence he himself entertains in the efficacy of the apparatus.

Albumen has been regarded as the most effectual antidote to Corrosive Sublimate. It is stated in a recent French Journal,‡ on the

* Extract of a letter from Dr. Von dem Busch, of Bremen, to Dr. Eberle, of Philadelphia, dated January 6th, 1822.

† *Toxicology*, vol. i.

‡ *Journal générale de Médecine*, Juillet 1822.

authority of M. TADDEI, that Gluten is to be preferred, as effecting the decomposition of the poison with much greater rapidity.

Some researches have been made by Mr. MURRAY, with a view of determining the counter-poisons of the Hydrocyanic Acid and Opium.* He had found that the violent head-ach sometimes brought on by preparing prussic acid, was always relieved, or entirely cured, by ammonia; and hence was led to regard this substance as the best antidote. This opinion was subsequently confirmed by various experiments: among others, half a drachm of hydrocyanic acid was given to a young rabbit, by which the respiration was rendered "laborious and difficult, with a grating in the throat; the eye lost its brilliancy; the head dropped; it raised a sharp cry, and was convulsed." Ammonia was now put into the mouth, which was likewise repeatedly moistened with a sponge dipped into a solution of the same: this had the immediate effect of reviving the animal, which appeared sensible of the relief afforded, by licking the finger with which the ammonia was applied. Such is the faith reposed by Mr. Murray in this antidote, that he informs us he "would feel no hesitation whatever in taking a quantity *sufficient to prove fatal*, provided there stood by a skilful hand to administer the remedy." Indeed, he did actually take a dose of the prussic acid sufficient to produce violent head-ach and stupefaction, which symptoms were instantly relieved by diluted ammonia, taken internally, and externally applied to the forehead and nostrils. Dr. GRANVILLE (to whom we owe the introduction of prussic acid into medical practice in this country) has likewise recommended the use of stimulants.

Mr. Murray recommends acetic (not acetous) acid as an antidote to Opium; but the discovery of the active principle of that drug in the form of *morphia*, and the fact of its increased energy by combination with vegetable acids, render his deductions on this point more questionable.

The extent to which even the most powerful medicines may sometimes be taken with impunity, is strikingly illustrated in a case related by Dr. FOGO, of the Royal Artillery.† A person labouring under an asthmatic complaint was ordered tincture of digitalis; of which the patient, being in great agony, and wishing to obtain relief at any hazard, swallowed an ounce at once. He fell asleep, and continued in that state for three hours and a half, when he awoke, and vomited "a good deal;" he had likewise an evacuation *per anum*. It was not discovered by his medical attendant that he had taken this immense dose of digitalis until more than six hours after it had occurred, when he immediately gave him a powerful emetic, which completely emptied the stomach. Nearly twelve hours elapsed before the pulse became affected, when it began to intermit and diminish in frequency,—a state of pulse which seems to have gone on for about twelve hours longer, having at one time fallen so low as thirty-six in the minute; but which speedily returned to its natural state, beating above seventy, and being free from intermission, twenty-eight hours after the digitalis had been swallowed. The treatment consisted in stimulants, particularly punch, ammonia, and nitric

* *Edinburgh Philosophical Journal*, No. xiii.

† *Edinburgh Med. and Surg. Journal*, July.

æther; and the patient not only survived the effects of the medicine, but even recovered from his pectoral complaint.

### MATERIA MEDICA.

Belladonna has occasionally been recommended in Tic Douloureux, and two cases of its successful exhibition have lately been published by Mr. THOMPSON, of Whitehaven. He gives it in doses exceeding what has been regarded as quite safe. "Perhaps, (says he,*) not less than two grains of the pure extract should be taken: oftentimes, when the pain is excessive, three or four grains, repeated at the end of five or six hours, and again till its action is manifested, are requisite,—watching minutely its progress. There are writers who say that it may be given to the extent of half a drachm, or more; but four or five grains not unfrequently, given in divided doses, will show effects sufficiently alarming to prevent a further continuance." The caution necessary in exhibiting this drug is sufficiently obvious in the cases related by Mr. Thompson: in the first, two grains and a half were taken on an empty stomach, which produced vertigo, faintness, and dimness of sight, to so great an extent as to render it necessary to confine the patient to bed next day; and she could not raise her head from the pillow for thirty or forty hours, "without experiencing a sensation she could not describe." In the second case, two pills, containing two grains each, were taken, which produced great vertigo, and even slight delirium; so that they were, of necessity, discontinued. In both instances, however, the pain was removed.

The property possessed by belladonna, and various other substances, of producing dilatation of the pupil when dropt into the eye, is known to all our readers; but we would call their attention to the fact that Stramonium seems to be equally powerful with belladonna itself, and therefore forms the most advantageous substitute for it.†

Some interesting remarks have been published by Mr. MONEY,‡ on the use of Oil of Turpentine. His observations are selected from clinical notes kept at the Northampton Hospital, from 1811 to 1816, and which comprise the histories of many cases. It was used in a variety of diseases, and proved beneficial in all, *except one*. The results given by Mr. M. are as follow:

"By the small doses, (as half a drachm, and less, taken repeatedly in the day,) obstinate chronic rheumatic pains have been removed.

"By doses of one drachm to two drachms, twice, thrice, or four times a-day, cures have been accomplished in adults labouring for two years under epileptic fits.

"In doses of six drachms every other morning, and continued for six weeks, children at the age of twelve years have been roused from a cloudiness of intellect, bordering upon idiotism of the melancholic kind.

"As a vermifuge, it has been given to the extent of one, two, three, and four ounces for a dose, the patient fasting: and its beneficial ope-

---

* *London Medical Repository*, July.

† *Annual Report of the Liverpool Institution for Diseases of the Eye*, by ALEX. HANNAY, M.D.—London Med. and Phys. Journal, September.

‡ *Medico-Chirurgical Review*, September.

ration has not been confined to the tape-worm, but all the species of worms have been alike expelled.

"In cases where several anomalous symptoms existed,—such as pain in the region of the stomach, distention of the abdomen, irregular bowels, slight but irregular paroxysms of fever, wandering pains, pulse full, and disinclination to labour, without any emaciation;—in these cases, in doses of two, three, or four ounces, taken fasting in the morning, it has produced the speediest and best effects."

Dr. GIBNEY has also recommended its employment in more liberal doses* than practitioners have been accustomed to. "There are few children of three years of age who will not bear from one to three drachms, given at intervals." He advises it to be given on an empty stomach, as little combined as possible with other remedies, and repeated at intervals of about an hour, for two or three times in succession, or according to circumstances: if its operation be delayed, a dose of castor-oil is recommended. This remedy also continues to have advocates for its exhibition in puerperal fever.†

Stramonium has been much extolled by Dr. ZOLLICKOFFER in the cure of rheumatism. He gives it in the form of tincture, made by macerating one ounce of the seeds in half a pint of spirits of wine, (spirit. vin. ten.) for seven days; after which it is to be strained, &c. and one ounce of laudanum, two ounces of the spirit. vin. camphor. and some aromatics, added. This compound tincture is asserted to be particularly beneficial in chronic rheumatism, given in doses of about eight drops morning and evening, gradually increased till it excites vertigo. He likewise uses the stramonium externally, in the form of ointment.

Prussiate of Iron is likewise among the candidates for admission into the materia medica, on the authority of Dr. Zollickoffer; and merits a trial, in the hands of those who prefer seeking after new remedies, to the use of those already known.

The same author has proposed the Podophyllum Peltatum, or *may-apple*, and the Apocynum Androsœmifolium, or *Canada dog's-bane*,—the former as a substitute for the jalap, and the latter for ipecacuanha: but why any thing should be substituted for two such efficacious and plentiful drugs, does not appear very evident.‡

Among the articles of materia medica which have lately attracted notice is the Sulphate of Quinine, for the knowledge of which we are indebted to M. DOUBLE,§ in some observations laid before the Institute of France. This substance has principally been employed as a substitute for cinchona, in cases where the exhibition of that drug would have been likely to produce inconvenience. Its medical efficacy is consequently most remarkable in the cure of intermittent and remittent fevers, although cases are recorded in which it has proved serviceable in neuralgia, and some other complaints, particularly scrofula. In hooping-cough, it seemed to do harm. The dose varies from four to twenty-four grains *per diem*; and, according to M. Double, it may be advantageously combined with calomel,—a grain of each every morning to

* *Edinburgh Med. and Surg. Journal*, No. 72.

† *Dublin Hospital Reports*, vol. iii. &c.

‡ *American Med. Recorder*, Nos. 17 and 18. § *Revue Medicale*, Mars 1822.

children; the same dose three times a-day to adults. The demand for this drug is so great in France, that we are informed by one of the medical professors of Edinburgh, lately returned from Paris, that Pelletier is in the habit of using thirty-six pounds of bark daily in its formation; this quantity yielding from thirteen to fifteen drachms of the quinine, which is sold at the rate of thirty francs per ounce.—Mr. GARDEN, of Oxford-street, informs us that, according to his manipulation, a pound of bark affords about three drachms: this proportion, it will be observed, agrees with that of the French chemist. We have exhibited the sulphate of quinine in a case of tic douloureux, during a fortnight, without benefit, in doses gradually increased from one to four grains four times a-day.

About twelve years ago an attempt was made, by M. CHREITIEN, to add gold to the list of the materia medica; and, although some trials were made by MM. BERTRAND, FODÉRÉ, and DESTOUCHES, in France, by HUFELAND in Germany, and by various physicians in America, yet the remedy seems, upon the whole, to have excited little attention, and gained but very few proselytes. A fresh attempt in favour of gold has recently been made by Dr. NIEL, of Marseilles, in a work extending to 392 pages. The effects of this new candidate for medical reputation are said to be immediate or direct, and definitive, efficacious, or therapeutic, ("definitive, efficace, therapeutique.") The direct effects are to exalt the vital functions, increase the pulse, and quicken the circulation; the appetite is augmented, digestion is improved, the excretions increased, and the whole system pervaded with an unwonted degree of vigour and hilarity. The second class of effects are of an "occult" nature, and, of course, not to be explained. As the stimulant properties are very active, it is regarded as a remedy requiring much caution in its exhibition. The triple muriate of gold and soda is said to be the most efficacious form, and friction upon the tongue one of the best means of introducing it into the system. The first grain is to be divided into fourteen frictions, the second into thirteen, and so on. It may likewise be given internally, in the form of pill, or used topically as an ointment. Ptyalism is one of the effects most worthy of notice, which is said never to produce the same inconvenience as that excited by mercury. Sometimes sweating is produced, which lasts for a fortnight or three weeks; but more frequently a diuresis supervenes. The diseases to be cured by gold are chlorosis, glandular swellings, scrofulous ophthalmia, fistulous ulcers, goitre, &c.—but its principal virtues are antisyphilitic. This appears to us the slenderest prop on which the pretensions of any medicine can be supported; for, in this country at least, we are too much accustomed to see venereal complaints cured by the powers of the constitution, assisted only by rest and proper regimen, to have any doubt of the accuracy of M. Niel in asserting that syphilis may be cured by the exhibition of gold,—or any thing else, combined with the auxiliaries mentioned above.*

M. LALLEMAND also advocates the efficacy of gold.†

---

* *Bibliothèque Medicale,* Août 1822.

† *Journal Universel des Sciences Medicales,* Août 1822.

### SURGERY.

We have now to relate what has been done within the last six months towards the perfection of Surgery, in doing which we shall adhere as closely as possible to the *art*,—including the performance of new or rare operations, the invention of instruments, or the perfection of any of the means by which those complaints which come especially within the province of the surgeon are more speedily and effectually removed. It must not be forgotten, however, that modern surgery claims a more extended field of action, and ranks high in our estimation as a science; yet, as it is impossible, and would certainly be improper, to restrict the application of the advances that have been made in physiology and pathology to the surgical department only, the majority of these have already been considered, and discussed in the former part of this Proëmium. Perhaps the circumstance which would most strike one of our surgical ancestors, if he could revisit the earth, would be the almost every-day occurrence of the operation for tying the largest arterial trunks. So rapid has been the advance of our art in this respect, that, within our own recollection, an hospital surgeon, now deceased, challenged the profession to produce even a solitary example of the successful result of the operation for popliteal aneurism. No six months now elapse without presenting us the history of many operations of even greater importance; and, within the period we are now about to describe, the records of the profession have been enriched with the details of some brilliant cases of this description. The carotid,* the external iliac,† the subclavian arteries,‡ have been respectively tied with success, and not in solitary instances only.

We will begin with the first of these arteries; and, in mentioning Dr. MOTT's bold and successful operation, we must beg to state that it is not quite so novel as he supposes, having been performed by M. GRAFE, of Berlin. In saying this, we by no means wish to detract from the merit of our transatlantic brother: on the contrary, we ardently hope to see an end to all those petulant and ill-natured remarks as to country, which so strongly savour of political hostility, and which, as men of science, we should know nothing of. Perhaps, it would be but candid, whilst expressing our regret that this ill taste should ever have been indulged, to observe how much that regret is heightened by our belief that this evil practice originated on our side of the Atlantic, and in a publication from which more liberality of sentiment might have been expected.—Dr. Mott was consulted by a lady, aged twenty-two, on account of an osteo-sarcoma of the left jaw, which had existed above a year, and extended, at the time he saw her, from the angle of the jaw to the first bicuspid tooth, being about the size of a hen's egg. On the 30th of March, Dr. Mott performed the excision of this tumor, having previously tied the carotid artery. The ligature came away on

* *American Medical Recorder*, July.
† *Medico-Chirurgical Transactions*, vol. xii.
‡ *Dublin Hospital Reports*, vol. iii.

the thirteenth day; the wound of the cheek healed, for the most part, by the first intention; and the patient returned to her residence on Long Island, entirely well, on the thirty-seventh day after the operation.

Dr. Mott performed the same operation on the 15th of May, tying the right carotid artery, and extirpating all the diseased mass, by sawing through the bone at the situation of the second bicuspid tooth of the left side, and then dissecting the bone from its articulation. The weight of this tumor was twenty-two ounces, and the size was that of a full-grown fœtus. The patient, however, died on the fourth day after the operation.

Of the Ligature of the External Iliac, we have two unsuccessful cases recorded by Mr. Todd; and a successful one by Mr. SALMON, of the Guards, and which, although performed upwards of a twelvemonth ago, we mention, because it is to be found in the last volume of the Transactions of the Medico-Chirurgical Society.

We now pass on to the Ligature of the Subclavian Artery, which has been tied by Mr. TODD, of Dublin, in a case of axillary aneurism. It is true that several cases of this kind are on record, most of them terminating fatally; and we are induced particularly to notice this operation of Mr. Todd's, because we consider his description of its various steps highly interesting, and detailed in the most perspicuous and satisfactory manner. The candid account he gives of the difficulties this case presented, and the improvements he suggests in any future operation, are highly deserving of our warmest praise, as well as our deepest attention; and we therefore conceive that we shall perform an essential service to our readers by transcribing his account of the operation.

The only facts previous to its performance, necessary to be known, are—that the man was fifty-three years of age, the disease had been no more than six months in making its appearance, and the tumor in the axilla was not detected by the patient until three months after he first experienced a numbness of the arm, and an inability of closing two fingers of the right hand. He entered Richmond Hospital on the 21st of January, 1822. At that period the tumor was very large, extending backwards so as to cause the scapula to project; it was also very prominent anteriorly, tense, elastic, and pulsating; the skin stretched, but not discoloured; and so large as to extend to the nipple of the breast, and on the side of the thorax to the upper edge of the sixth rib. There was no pulsation of the radial artery; great œdema of the limb, though but little diminution of temperature. The operation was performed on the 8th of February, in the following manner:

" The patient was placed on a table, lying on his back, with the upper part of his thorax somewhat raised; his head and neck inclined to the left, and his right shoulder as much as possible depressed by an assistant steadily drawing down the arm of that side. A slightly-curved incision was made through the common integuments across the lower parts of the neck, commencing about two inches above the acromial, and terminating half an inch above and to the outer side of the sternal extremity of the clavicle. The convexity of this incision was downwards, so that, by a little dissection of the integuments upwards, a small flap was made, which afforded ample room for the subsequent stages of the operation, and evinced the inutility of a more extensive or a more complicated division of the skin.

"The next part of the operation consisted in dividing the platisma myoides, fascia, and subjacent cellular tissue: this occupied a considerable time, in consequence of the great number of veins which it was found necessary to secure with ligatures. The external jugular, and two or three other superficial veins, were easily secured, but a series of more deeply-seated veins proved extremely troublesome: one branch of these, in particular, poured out blood in an alarming quantity, and receded so much within the layers of the fascia, that I was at last compelled to use the needle, and to include in the ligature the portion of fascia with which the divided vein was connected.

"I feel it incumbent on me here to state, that this profuse discharge of venous blood was chiefly the consequence of the veins having been divided too near the large trunk into which they opened; the blood, therefore, flowed freely, in a retrograde direction, from the subclavian vein into them, and issued from their inferior orifices: the bleeding from their superior orifices was inconsiderable, and easily controlled. To have tied these veins individually before dividing them, would have been an undertaking both tedious and difficult to execute, for they constituted a most intricate plexus of convoluted vessels, imbedded in cellular tissue and layers of fascia.

"The venous hemorrhage having been at last effectually suppressed, I proceeded to search for the omo-hyoideus muscle: so much, however, was the relation of parts altered by the magnitude of the tumor, and consequent elevation of the clavicle, that the portion of this muscle expected to be brought into view in this stage of the operation was situated more than an inch below the clavicle; and it was found necessary to draw it up from its concealment, and to cut it across, that the subjacent parts might become accessible.

"Having applied my finger to the edge of the scalenus anticus, I was directed by it to the situation of the artery; but at this juncture causes of further difficulty arose, chiefly from the great depth of the wound, and the doubt which the almost total absence of pulsation in the artery naturally excited in regard to its identity. It is necessary, however, to observe, that this obscurity in the pulsation of the subclavian artery was by no means referrible to the debility or exhausted state of the patient, but probably depended on the vessel having been flattened upon the first rib by the degree of extension to which the aneurismal tumor in the axilla had subjected it.

"For some time I could not be convinced that the feebly-pulsating vessel, to which the point of my finger was applied, was really an artery of such magnitude as the subclavian; and, aware of the disappointments which others were reported to have sustained in this operation, I resolved to satisfy myself and my assistants upon a point of so much importance, before a ligature should be applied. The depth of the wound rendered it impossible to see to the bottom of it; accordingly, I kept the point of my left fore-finger on the vessel, and cautiously detached it from its connexions with the blunt extremity of a director; having then introduced the fore-finger of my right hand also into the wound, I succeeded in compressing the vessel between the ends of my fingers, when the pulsation of the tumor immediately ceased,—returning when the pressure was discontinued. This expedient was conclusive, and, for obvious reasons, more satisfactory than that of pressing the artery downwards against the first rib.

"From the unusual degree of displacement of the clavicle, it was expected that great difficulty would have arisen in the application of the ligature to the artery; I was therefore provided with the several instruments which have been recommended to facilitate this step of the operation: however, none of these were employed, as the object was speedily effected with a common aneurism-needle. At first I attempted to pass the needle in front of the artery, with the view of giving every security to the vein; to this the position of the clavicle constituted an insuperable obstacle. I therefore directed the needle along the margin of the scalenus, and then insinuated the point of it under the artery from behind, guarding the vein with the fore-finger of my left hand, until the point of the needle was sufficiently elevated. I was then enabled to seize the ligature with the extremities of my fore-fingers, which I had introduced into the wound, nearly in the same manner as when compressing the artery; and the needle being held by an assistant, one end of the ligature was drawn out anteriorly, and the needle was removed.

"The artery then lay upon the ligature; and I requested that my assistants, and such other professional gentlemen as could conveniently approach the table, should convince themselves of this fact, by making the most accurate examination. The knot was now tied, and a sufficient tightness ensured by the ends of the ligature having been passed in the ordinary way through the serre-nœud. On the ligature being tightened, the pulsation of the tumor entirely subsided; its tension was considerably diminished, and the patient felt an increased degree of numbness of the arm. The external wound was then dressed, and he was laid in bed, with the limb supported on a pillow by his side."

We have in this place to mention an instrument which has been invented by Mr. Weiss, of the Strand, for the ligature of this artery; which, on account of the depth of the wound, and the relative situation of the parts being occasionally so much altered by the aneurismal tumor, is sometimes a matter of great difficulty, and is well illustrated in Mr. Mayo's interesting case,* in which the passing the ligature under the artery was not accomplished without much trouble, in consequence of the close adhesion of the vessel to the rib. After all, however, we find that he did succeed with Dessault's aneurismal needle, and that the operation altogether only occupied twenty minutes. For our own parts, we profess ourselves as decidedly hostile to the adoption of all mechanical contrivances in the performance of operations, that are not proved to be *absolutely* necessary; conceiving no instrument to be too simple for a good surgeon, whose finger is his best guide and assistant. We do not say this with any intention of depreciating Mr. Weiss's

---

* *Medico-Chirurgical Transactions*, vol. xii. part i.

invention: we have a high opinion of his ingenuity, and we shall have occasion to make a very favourable mention of him in the course of our observations. The instrument he recommends to our notice consists of a bent hook, the end of which contains a needle, armed with a ligature concealed in it; the extremity of the needle projects a little beyond that of the hook, and is caught by a little pair of pincers, which is guided to it by slipping it along a groove in the handle of the instrument. If the surgeon could be satisfied, by any other means than actually feeling the artery, that it alone was included in the ligature, this instrument might be of use: certainly, might get at the bottom of the wound when his fingers could not.

In close connexion with these operations is that which has been recommended for the cure of one species of Bronchocele, which has been proposed and practised in this country and on the continent. The case recorded by Mr. COATES* is the only successful one performed in England, we believe; and we have now to mention the favourable result of a second operation of this kind, performed by Prof. WALTHER himself. It is well known to the profession that this gentleman published observations upon this disease in the year 1817,† in which he describes a particular species of bronchocele, which he calls the aneurismal, and for which he recommends, and had practised, the ligature of the superior thyroideal artery. The following additional instance of success will be read with interest:

"John Otto, a locksmith, aged thirty-three years, a native of Linz, on the Rhine, had from his childhood a bronchocele, which gave him little inconvenience until he arrived at the age of twenty-five. He was born of healthy parents, and always enjoyed good health until he arrived at his thirtieth year, at which time he was first affected with a cutaneous eruption of the nature of psora, a gleety discharge from the urethra, and anasarcous swellings, which, joined to the tumor of the thyroid gland, ultimately obliged him to quit his employment. When I first saw him, his face was pale, and somewhat tumefied; the respiration difficult and obstructed. The tumor was tense, hard towards its centre, and pulsating strongly. The pulsations, equally with those of the carotids, were irregular, and not isochronous to the beating of the heart. Deglutition had become so difficult, that, in order to effect it, he was obliged to make extraordinary movements with the head and neck. In other respects he complained only of an extreme degree of snoring when asleep; but, while in a state of rest, he had neither head-ach, nor vertigo, nor epistaxis, nor any other bloody discharge.

"On the 2d of November, 1820, I undertook this operation, which was performed in the following manner:—After having divided the integuments and the thoracico-facial muscle of the left side of the neck, and after having exposed, at the distance of four lines from the superior margin of the tumor, the left superior thyroideal artery, I found it dilated to such a degree as to surpass the diameter of a common quill. Having passed under it, by means of a blunt curved needle, a silk ligature, without including any of the cellular tissue, I tied the artery in the

* *Medico-Chirurgical Transactions*, vol. x.

† *Neuve Heilart des Kropfes, &c.* 8vo. Sulzbach, 1817.

usual way. After the application of the ligature, the artery, below where it was placed, immediately ceased to beat, and became considerably contracted. I disposed the extremities of the thread to proceed from the wound in the usual way, applied straps of adhesive plaster, and covered the whole by a slight bandage. The patient was directed to incline his head to the side operated upon. An antiphlogistic treatment and mucilaginous drinks were ordered.

"During the first days after the operation, the patient felt as well as could be expected from the circumstances of his case. He complained only of a slight pain in the wound, and in the teeth of the left side. Deglutition, which before the operation was made with great difficulty, was now performed with facility. Traumatic fever appeared the third day, accompanied with a slight inflammation of the face on the side operated on, which required blood-letting to the extent of ten ounces, and the decoction althæa officinalis with nitre. The only unfavourable circumstance was the separation of the ligature on the eighth day, whilst a portion of the wound remained open, which discharged a sanguineous pus. As the ligature had divided the arterial tunics before re-union of the superior portion of the wound was sufficiently firm to support the extremity of the artery, and resist the afflux of blood, and as the pulsation of both carotid and thyroideal arteries were very strong, I dreaded that consecutive hemorrhage might supervene. To prevent this accident, I enjoined absolute rest, a light and antiphlogistic diet, and prescribed the hydrocyanic acid in order to moderate arterial action. At the end of seven weeks, the wound was entirely cicatrized; deglutition and respiration were performed without any difficulty; tumefaction of the face had disappeared; the left half of the thyroid tumor, which before the operation was much more voluminous than the right, was entirely dissipated and reduced to its natural size; the pulsations of the left thyroideal artery could be felt, until it reached the place at which the ligature had been applied. This individual quitted the hospital quite cured."*

Whilst upon the subject of bronchocele, we must observe, that some very respectable additional testimonies of the efficacy of Iodine in this complaint are to be found in the latter Numbers of this Journal.†

Bronchotomy has been successfully performed by Dr. Hunt,‡ of Dartmouth, in the case of a boy, aged four years, who fell with some pebbles in his mouth, which were drawn into the rima glottidis, and subsequently passed into the trachea. There is nothing remarkable in the operation itself; but the symptoms which afterwards occurred appear to have been met in a highly bold and energetic manner, and are worthy of attention. The account of this case is enriched by some observations of Mr. Earle's, who strongly urges the early performance of this operation in similar cases, notwithstanding the apparent calm that sometimes ensues; and of which we do not know a more instructive instance than that recorded by M. Louis, in the Memoirs of the French Academy of Surgery, in which the poor patient (a child seven years of age,) was sacrificed to this erroneous opinion.

---

* *London Medical Repository*, October.

† *London Med. and Phys. Journal*, September and October.

‡ *Medico-Chirurgical Transactions*, vol. xii. part i.

It appears that Mr. Earle was mistaken in supposing that no other successful case of bronchotomy has been published in this country. Mr. WHITLEY,* of Halton, Cheshire, having by this means extracted a damson-stone from the trachea of a boy, a few years ago, and who is now alive and in perfect health.

Large Sarcomatous Tumors.—The records of surgery abound with descriptions of enormous fatty tumors that have been dissected from various parts of the human body. The following late instance, however, exceeds any thing of the kind we ever remember to have met with, although Sir A. COOPER once removed from the abdomen of a man a tumor weighing as much as the largest of the eight which were met with in the same subject in the instance here recorded.† The patient was a female, named Emilie Leue, eighteen years of age. The posterior part of the trunk of her body, towards the cervical region, presented two tumors, eight inches long and three broad, uneven, covered with small whitish spots; a third, near the arm-pit on the right side, was very small, round, and soft; the fourth, situated beneath the inferior angle of the right scapula, was one foot three inches long, and six inches broad; the fifth, which was situated immediately below the preceding, was six inches long and five broad; the sixth was larger than a man's head, and arose from the external part of the hip-bone; the seventh was near the trochanter of the same side, and was much smaller; and the eighth had its origin at the left hypochondrium, and reached down as low as the calf of the leg, being two feet long, and three feet one inch round its base. All of them were isolated from the internal organs and muscles. M. DAGORN, (the operator in this case,) believing these tumors to be confined to the integuments and cellular membranes, considered their amputation practicable. The operation was performed on the 20th of July, 1819, upon the largest tumor, which weighed forty-six pounds. The wound cicatrized at the end of little more than two months; after which the other tumors increased in size considerably.

We shall do no more in this place than mention Sir A. COOPER's splendid volume on Dislocations, the plates of which are not only well executed, but, we should conceive, must alone have cost to the full amount charged for the volume: they are thirty in number; and the work itself forms a complete treatise on these accidents, and which should have a place in every surgical library. We shall take an early opportunity of making our readers acquainted with the contents of this volume.

On the subject of Fractures, we have to notice some cases of compound fracture related by Mr. DUNN, of Scarborough,‡ and a paper by Mr. AMESBURY,§ on what he terms a new apparatus for the treatment of fractures of the lower extremity.

The cases of Mr. Dunn have produced some discussion, and we shall therefore say a few words respecting them in this place. We consider

* *London Med. and Phys. Journal*, November.
† *Revue Medicale*, Août 1822.
‡ *Medico-Chirurgical Transactions*, vol. xii.
§ *Quarterly Journal of Foreign Medicine and Surgery*, July 1822.

them valuable as proofs of what nature is able to effect under circumstances apparently the most discouraging, but not as models for the young surgeon to follow. In saying this, we mean no reflection upon Mr. Dunn's practice; for we think it no more than fair to allow him, who was upon the spot and master of all the circumstances, to be, at least, as good a judge of what ought to have been done, as those who only know the facts through the medium of the press. But we conceive that the *general* attempt to save limbs so fractured, and to stitch together lacerated wounds of such extent, would in the main be bad practice, since the chances of recovery are very few. The limb, if saved, proves so often to be an incumbrance instead of a benefit to the patient,—liable to ulceration, to œdema, to violent periodical pains, and with the muscular motion very limited and imperfect,—that we consider the majority of those who are doomed to labour for their support would infinitely prefer a wooden leg: and indeed, in higher life, we have seen more than one instance in which the sufferer has bitterly lamented the success of his surgeon in saving a limb worse than useless,—a constant source of uneasiness and trouble. Still, however, we are of opinion that, if ever the attempt to save limbs so lacerated be justifiable at all, it was certainly so in these instances, in which the youth of the patients was a circumstance highly encouraging. The third case, of amputation of a projecting edge of the tibia, after the union of a simple fracture, presents no novelty, and certainly nothing to object to.

Mr. Amesbury's paper on Fractures is in many respects deserving of attention. We despair of being able to afford our readers an idea of the mechanical contrivance which he recommends, as the description occupies above three pages of letter-press, and there is no diagram to assist the elucidation; but the following indications, which he thinks should be always borne in mind in the construction of any machine for fractures of the lower extremity, will put our readers in possession of the principles upon which his practice is founded:

"1. It should fix the whole limb, so as to admit of no motion whose centre is not in the hip-joint, or between the pelvis and the back. 2. It should maintain the fractured ends in a natural position, and in perfect co-aptation. 3. It should lie upon the limb with ease to the patient. 4. It should enable the surgeon to place the limb at any angle the case may require. 5. It should allow of the application of extension and counter-extension, when the limb is in the bent position. 6. It should be entirely passive to the motions of the limb, and should allow the patient to place it in any position most congenial to his feelings, either on the heel or on the side, and to alter this position at pleasure. 7. It should enable the attendants to move the patient from place to place, without any danger of displacing the fractured ends. 8. It should allow of being adapted to limbs of different lengths and different sizes. 9. It should be applicable to fractures in any part of the limb, and of all kinds, whether simple, comminuted, or compound. 10. It should be simple, and easy of application. 11. With all these advantages, it should ensure to the patient a speedy recovery, and a straight and perfect limb."

Of this we are quite convinced, that all the contests as to what position a fractured limb should be placed in,—whether straight or bent, outwards or inwards, elevated or not, are futile: the surgeon must be sufficiently master of mechanics to be able to apply his powers to the nature of the individual case; and, probably, no given number of fractures, either of the leg or thigh, can be treated precisely in the same manner, with regard either to position or apparatus.

A case of re-union of the broken ends of bone is to be found in the American Medical Recorder for the month of July. Dr. DUCACHET succeeded, by means of a seton passed between the extremities of the fractured radius, to cause a bony union to take place in about two months. The fracture had existed rather more than ten months. This case is interesting, because we have, within the same period, the relation of some cases by Mr. H. EARLE,* in which the same measure, adopted under circumstances apparently favourable, failed to produce a like fortunate result.

A suggestion respecting the possibility of destroying Cancer by the application of intense cold, has been started,† and which seems to have originated from a consideration of what VAN SWIETEN, in his Commentaries, has hinted at in these words,—"Sed an non possit totus cancer emori:" but, though we do not doubt the possibility of inducing mortification by means of metallic plates artificially cooled, yet we are of opinion that it would be a very bungling and painful method of arriving at the same result which the knife would produce in a few minutes, and with the advantage of saving as much skin as necessary. Nor are we prepared to say that the application of such intense cold to the human body might not be productive of constitutional mischief, not much less serious than the complaint it is proposed to remedy.

On the subject of cancer, a memoir of the celebrated SCARPA's has just made its appearance;‡ it was inserted in the last volume of the Transactions of the Imperial Institute at Milan, and is translated by Mr. BRIGGS. The principal doctrine advocated appears to be the local origin of scirrhus; but we shall give a more detailed account of this pamphlet in one of the succeeding Numbers of our Journal.

An operation not absolutely new, but which is certainly remarkable enough both for its rarity and boldness, has, within these few weeks, been performed in one of our city hospitals, upon a patient who had been run-over, on the trunk of the body, by a heavy cart, which fractured and displaced two of the vertebræ. The operation consisted in cutting out the posterior portion of these vertebræ, (which were occasioning pressure upon the medulla spinalis,) together with the spinous processes, thereby exposing the spinal canal the whole length of the bodies of the two bones. The patient died; but his death appears to have arisen from the complicated injuries resulting from the accident, and not to have been at all dependant upon the operation itself. This practice has been recommended by BOYER§ and other authors, and practised by Mr. H. CLINE; but we scarcely think it justifiable, because, al-

* *Medico-Chirurgical Transactions*, vol. xii.

† *Philadelphia Journal*, No. vi.

‡ *Observations on Scirrhus and Cancer, &c.* Cadell, 1822.

§ BOYER *Traité des Maladies Chirurgicales.*

though pressure may be removed by it, it can scarcely be expected that the spine can ever again become useful in the erect posture; not only from the great loss of bone, but from the destruction of the attachment of so many muscular fibres: so that the patient would, in all probability, if he lived at all, be condemned to the horizontal posture during life,—a situation hardly to be preferred to death itself.

A case of Excision of the Neck of the Uterus is given to us by Mr. AVISARD.* The circumstances were the following:—At thirty-seven years of age, the woman contracted syphilis, and a mercurial course of some length was followed by epilepsy. In 1819 (seven years after,) a sense of heat was felt in the pudenda, with darting pains in the loins and thighs, and general ill health. In 1820, on examination, the orifice of the urethra was found to be of a red violet colour, and excessively tender; there was no discharge, but the neck of the uterus was swelled and hard, especially on the fore-part, but not ulcerated. The body of the uterus was healthy. On March 23, 1820, the patient being placed on a bed in the position for lithotomy, the neck of the uterus was brought forwards by the fore finger of the right hand. M. DRECQ pressed on the hypogastrium; and a double hook (such as is used to raise parts in dissection,) was passed into the os uteri as far as the thickening of its upper lip extended. The neck of the uterus was then slowly drawn to the mouth of the vagina, and cut off by strong curved scissars. The part cut off was as hard and almost as white as cartilage, and its diameter equalled that of a five-franc piece. The upper part, which answered to the anterior part of the os uteri, was seven lines thick; the lower part was five lines thick. The slight hemorrhage was diminished by injections of oxycrat; the vagina was stuffed, and a bandage applied. The patient was placed in bed, with her head raised, and her legs and thighs bent on her pelvis. After some fever and other symptoms of abdominal derangement, the patient was examined again on the twenty-first day. A speculum uteri was employed; and a small projection at the back part was found, and removed in the manner above described: but now nitric acid was applied to the cut surface, by means of charpie, on the thirty-seventh day. This was repeated, a portion of a small enlargement still remaining; but, being soft, it tore, and nitric acid was again applied. Some weeks after, another hardened portion was cut off, and the acid applied two or three times. Nine months after the operation, the os uteri was found to be white, and only a little hard. The patient continued well for twenty-seven months after the operation, and has had no attack of epilepsy. She has some slight uterine pains at the menstrual periods.

Two instances of Inverted Uterus, successfully treated by ligature, occurred in the practice of Dr. CHARLES JOHNSON.† In both these cases, the tumor which came away proved to be the fundus of the uterus and the fallopian tubes. In one of the cases, the application of the ligature produced much constitutional disturbance; but in both the recovery was complete.

---

* *Journal Universel des Sciences Medicales.*

† *Dublin Hospital Reports*, vol. iii.

The Cæsarean Section has been performed by MM. HORN, father and son, on the wife of one Amos, of Fischelbach;* and they had the satisfaction to save both the mother and twin infants. The operation was rendered necessary by a firm adhesion of the superior part of the vagina to the os uteri. Between the labia majora there was an aperture of not more than an inch in extent, and which with great difficulty would allow the immisio penis. After recovering from this operation, the patient lived in her usual state of health for nine months, and also the twin infants, who had been recommended to a good nurse.

Mr. GREEN† has given us an account of his having performed the Cæsarean section upon a woman, who was run-over by a stage-coach in the Borough, and immediately conveyed to St. Thomas's Hospital, where she expired in twenty minutes. Dr. Blundell and Mr. Green agreed on the propriety of performing this operation, which was done within a quarter of an hour from the death of the mother. The child, when extracted from the uterus, gave no signs of life; but, by inflating the lungs for about fifteen minutes, respiration was established, and at the end of fifty-two minutes the child opened its eyes: it lived, however, only thirty-four hours. This case is interesting and encouraging, and the operation should never be lost sight of or omitted under similar circumstances.

Sir A. COOPER has presented us with an instrument for dilating the female urethra, and which he has successfully employed in two cases, detailed in the last volume of the Transactions of the Medico-Chirurgical Society. In the first case, the dilatation was continued for some hours, and a severe attack of irritative fever followed the operation. In the second case, the extraction of the stone was performed in a few minutes; the extension being made, however, very gently. In this case there was incontinence of urine for a short period; in the first instance, the power of retaining it was never lost. Sir A. Cooper thinks that further experience will be required to determine if it will be best to dilate the meatus in a few minutes or hours, or in several days. If the stone be small, he thinks the operation may be performed in a few minutes; but, if large, it may be better to dilate a little from day to day, doing so carefully, avoiding contusion, *which is much to be dreaded.* The instrument will be best understood from the engraving, but the following description will give a tolerably accurate idea of it:—It consists of something like the blades of a straight forceps, which are introduced into the urethra closed. They are hollowed, and between them is placed a stile, which, by means of a screw in the handle, can be drawn back upon the inclined plane of the blades, and consequently made to force them open. This instrument was constructed by Mr. Weiss, of the Strand, to whom Sir A. Cooper very handsomely assigns the merit of the suggestion.

A few remarks may, perhaps, be permitted us in this place upon the treatment of Gonorrhœa and Syphilis, in which the fluctuations of opinions and practice have been, within these few years, very remark-

* HUFELAND's *Journal der Practischen Heilkunde.*

† *Medico-Chirurgical Transactions,* vol. xii.

able. In the former of these diseases, we believe that all modern surgeons have totally abandoned the use of mercury as a means of cure, or as a mode of preventing the sequelæ, real or supposed, of the complaint; but there is still great difference of opinion as to the value of a remedy of more modern date—the Java pepper, the employment of which is most warmly advocated by two surgeons of great respectability,* and is represented as almost deserving the name of a specific:† whereas, more recently, Mr. CHURCHILL‡ has thrown out some doubts as to its efficacy, although he admits that his experience of its virtues is too limited to allow him to speak decidedly on the subject; but quite sufficient to deter him from using it while other modes of cure are to be relied upon. For our own parts, we consider the cubebs as a valuable addition to our curative means; but we have certainly seen very unpleasant symptoms arise during its use in the inflammatory stage of the clap, which is directly contrary to what Mr. Broughton has said; but we have also witnessed the most marked and striking good effects from the same remedy, given when the inflammatory symptoms have subsided, on more than one occasion putting an end to the discharge in twenty-four or thirty-six hours. Upon the whole, the evidence we have to give is in favour of the cubebs; but, certainly, we should be *almost* afraid to prescribe it in that stage of the malady where inflammation has become fairly established, though Mr. Broughton may be justified in giving it within the first forty-eight hours: and, indeed, it not unfrequently happens that even a stimulating injection, used prior to the establishment of the ardor urinæ, &c. will effectually cut off the disease at once.

On the subject of Syphilis we have a few novelties to notice, the first of which relates to the efficacy of the preparations of gold, of which so much has been said in France, by M. CHRESTIEN,§ of Montpellier, and more recently by M. LALLEMAND.|| The preparation in which he places most confidence is the triple muriate of gold and soda. This is introduced into the system by friction on the tongue, beginning with the twelfth or fifteenth of a grain. Great caution is required in its exhibition, we are told, as it is apt to disagree with irritable constitutions. The effect is always that of slight febrile excitement; perspiration follows, with an increased flow of urine; and also, though not so commonly, a flow of saliva, quite inodorous, and without any of the inconveniences of a mercurial ptyalism. We think that the value of this medicine is very much decreased by the certainty that now exists of our being able to cure all sores on the genitals by simple means only; and the observations of M. Lallemand tend to confirm this opinion, inasmuch as the cases he has detailed are, in our opinion, any thing but fair specimens of the venereal disease; and we have no knowledge, in this

---

* *Observations on Cubebs*, by H. JEFFREYS; and Mr. BROUGHTON's paper, *Medico-Chirurgical Transactions*, vol. xii.

† *London Med. and Phys. Journal*, October.

‡ *London Med. Repository*, October.

§ *Recherches et Obs. sur les Effets des Preparations d'Or de* Dr. CHRESTIEN. Par J. G. NIEL, &c. 8vo. Paris.

|| *Journal générale des Sciences Médicales*, Août 1822.

country, of such cases as the following, in which "the venereal virus, being imperfectly destroyed by a superficial treatment, remained latent in M. de C.'s constitution for several years, to be roused again (so to express it,) some months after marriage, and to show itself in the organs which were most excited. Communicated to Madame de C. it was again combated," &c.

However, we by no means wish to condemn the remedy without affording it a fair trial; but, for our own parts, notwithstanding all that has been said or written to the contrary, we are quite satisfied with the cautious and discriminate employment of mercury in the treatment of both primary and secondary cases of the venereal disease. This leads us to the consideration of Dr. Hill's paper* on the Simple Treatment of Syphilis, which, however well arranged and written, cannot be said to offer any new views of the subject. We have had now for these few years past a host of evidence from Messrs. Rose,† Guthrie,‡ Bacot,§ Thompson,|| Hennen,¶ &c. all proving the points which Dr. Hill has again presented to our notice; but from which we have been induced to draw conclusions somewhat different, inasmuch as we consider the general adoption of the non-mercurial practice as by no means safe or judicious, for the following reasons:—1. That sores, generally, do not heal so quickly without mercury as with it; 2, that eruptions, and other consequent symptoms, are infinitely more common when that remedy is omitted; and, 3, that though these may, and do frequently, get well by simple treatment only, the cure under those circumstances is very tedious, relapses frequent, and, in short, such as would by no means satisfy that class of patients to whom time is of any value. The common soldier, or the inmate of an hospital, may perhaps be prevailed upon to forget or to despise a crop of eruptions, the stains of which he is content to carry about him for months, or even longer; but such practice, although eventually leading to the restoration of health, would scarcely be submitted to by any other class of society, and nothing can be more certain than that the majority of these eruptions is under the influence of mercury, exhibited with caution and judgment. The great good that has arisen from our non-mercurial practice appears to be, that we do not now fly to mercury for every sore we meet with in the genitals or on the tonsils, and especially the latter. We acknowledge more universally the constitutional origin of local diseases; we feel the pulse carefully, and examine into the condition of the health generally, before we give mercury;—precautions which, we venture to say, were not adopted in one case in an hundred twenty years ago.

M. Lagneau's paper, with the observations of the writer in the Quarterly Journal for October, are well worthy of perusal, as they tend to confirm some of the views we have merely hinted at.

The inflammatory chancre has been noticed in the Number of this

---

* *Edinburgh Med. and Surg. Journal,* October 1822.
† *Medico-Chirurgical Transactions.*
‡ *Medico-Chirurgical Transactions.*
§ *Observations on Syphilis.*
|| *Edinburgh Journal* and *Medico-Chirurgical Review.*
¶ *Edinburgh Journal.*

Journal for August. The distinctions there laid down are not illusory; the discrimination of those cases from the simple sloughing chancre are founded in truth, and of the greatest practical importance.

There are some trifling points in the performance of common operations which yet remain to be noticed; one of which is a mode of extirpating the testicle by M. AUMONT,* who advises the scrotum to be lifted up, and laid upon the groin of the healthy side. The incision is made upon the posterior part of the scrotum, and carried upwards to the ring. This appears, to us, to be a change without an improvement.

M. DELEAU† recommends puncturing the tympanum in deaf-and-dumb persons. It has not yet been attended with much success, but the operation, in its application at least, appears, to us, to be new.

The last six months may be said to have been remarkably barren in new medical works of all kinds, but especially in the department more immediately under our consideration. Mr. WARD's work on Curvatures of the Spine, and Mr. BINGHAM's on Diseases of the Urinary Organs, are the most recent as well as the most prominent, with the exception of Sir A. COOPER's great work, which we have previously mentioned; and we do no more than name them here, as our intention is to give an extended account of them in future Numbers of our Journal.

### MIDWIFERY.

A new application of the stethoscope invented by LAENNEC, with particular reference to the sounds emanating from the chest, has been suggested by M. LEGUMEAU DE KERGARADEC,—viz. for the purpose of ascertaining the life of the fœtus in utero, and other circumstances connected with utero-gestation.‡ According to the author above mentioned, on applying the instrument to the region of the uterus, two distinct pulsations can be distinguished,—one being communicated by the heart of the fœtus, the other by the arteries of the mother: the former is double and more frequent than the latter, which is simple and synchronous with the pulse of the parent. It is likewise asserted, that the sound emitted by the arterial pulsations in the uterus vary to such an extent, according as the instrument is applied to different parts of it, that the situation of the placenta may be discovered, —the sound being most distinct when the stethoscope is applied over it. To this, however, M. FODERA objects, principally on the ground of experiments. He placed the instrument on the left side of the uterine region in a woman in the seventh month of pregnancy, when he perceived a pulsation synchronous with that of the arteries of the mother; in the median line the sound was lost, but was again perceptible in the right side, though less distinctly than in the left. It may be objected, that there might have been two placentas; but this was proved not to be the case.

The subject of Uterine Hemorrhage has received some illustration

---

* *Journal générale de Médecine*, Juin.

† *Journal Complémentaire*, Juin.

‡ *Memoire sur l'Auscultation, appliquée à l'Etude de la Grossesse, &c. &c.* Par M. L. DE KERGARADEC.

from the observations of Dr. Gooch, published in the last volume of the Medico-Chirurgical Transactions. He has observed two circumstances, in which bleeding may occur to such an extent as to prove alarming, "although the uterus feels contracted in the ordinary degree." The first of these depends on the constitution of the patient, —some persons having a peculiar proneness to fainting; and, consequently, if an individual so situated lose rather more blood than usual on the separation of the placenta, it may affect her as much as a more profuse loss of blood would another. Sometimes this liability to syncope does not depend upon constitutional tendency, but arises from an exhausting labour; in which cases it is attended with "nervous irritation," and requires opiates. The second circumstance is where, after delivery, the uterus contracts so as to close the blood-vessels against the impetus of *ordinary* circulation; but, the impetus being *extraordinary*, the contraction of the mouths of the arteries is overcome, and hemorrhage ensues. Dr. G. delivered, of her second child, a lady, who for many hours before the accession of labour had been flushed, and whose pulse was very full and quick. "Abstinence from meat, wine, and warm drinks, a cool room, and a saline purgative, diminished, but did not remove, this state of the circulation, which continued in a considerable degree when the child was born. It was expelled very gradually; and, after the removal of the placenta, the uterus fell on the hypogastrium, and contracted in the ordinary degree: nevertheless, about twenty minutes afterwards, there came on one of the most frightful hemorrhages I ever witnessed. By the introduction of the hand, and the application of cold, however, it was speedily arrested." The lady became pregnant again, and the same circumstances recurred, the hemorrhage being even more violent and alarming. She became pregnant a third time, and precautionary measures were adopted: these consisted in avoiding fermented liquors,—taking meat only three times a-week,—keeping the bowels open by the occasional exhibition of senna and salts, and a scruple of nitre three times a-day. This regimen was continued for two months before her confinement, yet the hemorrhage returned, although in a much less degree, and was easily checked. Having become pregnant once more, the same plan was adopted, with this addition, that twelve ounces of blood were taken from the arm about a fortnight, and eight ounces a few days, before the accession of labour was expected. No hemorrhage now occurred. The opinion of the ingenious writer is, that, although the ordinary contraction of the uterus may not be sufficient to prevent the blood from bursting forth, yet that an extraordinary contraction may; and that this is best effected by cold applied with a shock. The first time he attended the lady whose case is related above, the bleeding returned again and again, although the abdomen was covered with pounded ice; the uterus, at the same time, becoming so soft and relaxed that it could not be felt. The ice was removed, and the contents of an ewer of cold water allowed to fall upon the abdomen from a height of several feet: the uterus immediately contracted, becoming small and hard; while, at the same time, the hemorrhage ceased, and the faintness went off.*

* *Medico-Chirurgical Transactions*, vol. xii.

Another practical lesson is contained in the paper of Dr. Gooch, with regard to the introduction of the hand into the uterus to stop hemorrhage. He advises making an attempt, in such cases, to apply pressure directly upon the bleeding vessels. When any necessity for this can be anticipated, the exact situation of the placenta may be ascertained, before its removal, by running the fingers up the cord, and observing its direction; but, when this has not been the case, the pressure ought to be applied to the fundus, as the most likely point of attachment. This is to be done by introducing the left hand closed within the uterus, and applying the right hand open to the outside of the belly, and then pressing between them the part to which the placenta was attached.

The natural tendency of rupture of the membranes, and the escape of the liquor amnii, at whatever period of pregnancy it may occur, is to excite the uterus to contraction, and so to expel its contents. Some observations of interest, with cases in which labour did not come on till the full period of utero-gestation, have been published by MM. Maunaury and Léveque-Lasource.* In one of these, a lady aged twenty, of sanguine and nervous temperament, received, in the sixth month of her pregnancy, a very severe fall, in which the head and lumbar region were the parts most injured. Immediately after the accident, the movements of the child were very strongly felt, but soon entirely ceased; while the patient experienced pains in the thighs, and a sense of weight in the pelvis. During the night, a fluid, resembling in its physical qualities the liquor amnii, was discharged *per vaginam*, to the extent of about two ounces; and, at two in the morning, pains, supposed to be those of labour, came on. By absolute rest, however, this was averted; but it was not till fifteen days after the accident that the movements of the child were again perceived. It was born perfectly healthy at the full time. In another instance, a young lady, in the fifth month of her first pregnancy, used considerable exertion in opening some windows, by which she was somewhat fatigued, but not so much as to prevent her from going next day to the theatre, (she was a French woman,) where she was taken with flooding, and carried home, suffering severely from pain in the lumbar region and fits of syncope. These were followed by the discharge of about four ounces of what M. Lévêque-Lasource, her attendant, regarded as the liquor amnii. She was bled, and kept perfectly quiet in bed for three weeks; and it was not till five weeks after the accident that the movements of the child were again perceptible. The accouchement took place at the usual period of utero-gestation, and the child was well formed, but the action of the lungs was very weak, and the vital functions appeared to be in a state of "stupor and inertness:" nevertheless, the infant, with the benefit of much care and a good nurse, flourished in a manner "qui n'a rien laissé à desirer."

A case of Perforation of the Perinœum is recorded by Dr. Douglas.† A young woman, in the Lying-in Hospital at Dublin, was taken in labour of her first child, rather unexpectedly. When Dr. D. arrived, he

* *Bibliothèque Medicale*, Avril 1822.

† *Dublin Hospital Reports*, vol. iii.

found that the head had protruded, and was closely applied to the inner part of the left thigh, inclining backwards, and by a fresh pain the child was expelled. On examination, a lacerated opening was found, comprising part of the perinœum laterally, the integuments of the thigh, and running up into the left labium pudendi. The funis was introduced into the vagina, and brought out at the os externum; but the placenta chose, notwithstanding, to follow the child, and the after-birth was finally extracted by the wound. No bad consequences followed this accident, except that, as the part by which the infant had passed did not seem disposed to fill up sufficiently by granulation, and the sphincter vaginæ on that side wasting, it was divided. The writer conjectures that the fœtus burst through the cervix uteri, and passed down behind the vagina, near the sacro-iliac synchondrosis.

### CHEMISTRY.

Some new phenomena connected with Heat have been discovered by M. Pouillet.* Having been struck with the development of electricity, and the relation which subsists between the electric properties of a body and those of its elements, he was induced to examine the compounds of which water forms a part, whether as an essential element in a fixed proportion, or as an accidental expedient in variable quantity. Numerous experiments, made on simple bodies, as oxides, and more compound substances, (as glass, porcelain, and clay,) have led him to the conclusion, that caloric is disengaged at the moment when a solid is moistened by a liquid. This circumstance, if it shall be proved to exist as a general law, is obviously one of extensive application, and calculated to explain many phenomena, the nature of which has hitherto escaped the detection of the most acute philosophers. The act of moistening is one of constant occurrence: the rain which falls on the earth moistens its surface and the subjacent strata; vegetables have a source of heat in the same cause; and in the animal body, where various fluids are in constant circulation, the act of moistening is carried on at every moment, whether these fluids simply pass along the sides of the vessels, or pass through them by exhalation or absorption.

The quantity of caloric disengaged in the act of moistening, as shown by the experiments of M. Pouillet, is small, as indeed might be expected from the fact having hitherto escaped observation. This, however, does not lessen the importance of his discovery: for, in the physical world, the greatest natural causes are not those which act on matter with the greatest violence, but those whose operation is the most universal and the most constant.

The quantity of caloric disengaged being so small, requires extreme sensibility in a thermometer capable of measuring it; and, after trying the calorimeters of Leslie and Count Romford, M. Pouillet invented a mercurial thermometer, so minute in its divisions as to mark the hundredth part of a degree of the centigrade. Furnished with an instrument of this delicacy, he was able to establish that, when a piece of glass was moistened with water, caloric was evolved at the point of con-

---

* *Annales de Chimie et de Physique,* tom. xx.

tact, which communicated itself from this point to the glass, on the one hand, and to the water, on the other. But, by diminishing the number of the superfluous particles, which were heated at the expence of the others, and augmenting the proportion of surface brought into contact, he was able proportionally to increase the degree of heat. Thus, if, without changing the mass, he gave it a surface an hundred times more extended, it disengaged, when moistened, an hundred times more caloric. It is necessary, in order to render these experiments successful, that the proportion of the mass to the surface be kept in view; because, where the surface is not extended sufficiently, the disengaged heat is taken up by the mass so rapidly as to prevent it from becoming sensible. Thus, if a thick piece of glass be moistened, no effect is perceptible; but, if a very thin plate be used, the thermometer indicates a very sensible increase of heat. Another state in which bodies are capable of indicating these phenomena, is that of fine powder, by which the capillary attraction for the fluid is favoured, and the surface of contact extended. In this manner various metals, oxides, earths, and compound bodies, were used: the liquids were distilled water, oil, alcohol, acetic æther, and volatile oil of turpentine. The same general result presented itself,—viz. increased temperature, varying from one-fifth to one-half a degree of the centigrade thermometer; the elevation being nearly the same from different solids with the same liquid, and the same solid with different liquids.

Some experiments have been made by M. VAUQUELIN on the combination of acetic acid and alcohol with volatile oils.* Various proportions of the above acid and volatile oils were placed together in phials, and shaken, by which their union was effected; and it was found that, when the acetic acid is pure, the oil can absorb it entirely; but, if it contain any water, though not exceeding five per cent., a part will remain, which the oil cannot take up, so that the remaining acid, which does not enter into the combination, contains necessarily a greater proportion of water than vinegar before the process. These experiments agree with the facts already known of vinegar becoming impregnated with the odour of plants.

From similar experiments, it was found that one hundred parts of volatile oil of turpentine, mixed with twenty parts of alcohol, formed a homogeneous fluid, which did not separate on being left to settle. This mixture, or rather solution, is not disturbed by water; but, when poured into water, and gently shaken, a portion of the alcohol detaches itself, and unites with the water, forming distinct streaks.

M. DOEBEREINER† has made known the artificial formation of the Formic Acid. Tartaric acid or supertartrate of potass, peroxide of manganese, and water, are mixed together, and heated, by which means a violent action is excited, and a great quantity of carbonic acid disengaged. At the same time a liquid acid is distilled, which at first might be mistaken for *acetic*, but which closer examination shows to be *formic* acid. This acid, when mixed with strong sulphuric acid, changes

* *Annales de Chimie*, Mars 1822.

† *Annalen der Physick*, lxxi. p. 107.

at a moderate temperature into water and oxide of carbon; the nitrate of silver and the nitrate of mercury convert it into carbonic acid, by means of a gentle heat, the two oxides being reduced to the metallic form. M. Doebereiner is of opinion that formic acid is produced in several other processes,—as in treating sugar, alcohol, and other vegetable productions, with nitric acid; and, perhaps, even in many plants, by spontaneous formation. These experiments have been repeated by the editors of the Annales de Chimie, with results exactly similar.

A new application has been made of the Thermometer by M. F. GROENING, of Copenhagen. He was in the habit of using this instrument (in an apparatus for distilling, invented by himself,) with a view of ascertaining the temperature in the receiver. In the course of these experiments, he observed that the thermometer always rose to a certain point before any of the distilled fluid made its appearance, and that it continued at this point till about half the fluid in the retort was evaporated, when it rose, at first slowly, and then more rapidly. This phenomenon was at first supposed to depend upon the different temperatures of the spirituous and watery vapours; but, by further observation, he discovered that, as long as the thermometer remained at a fixed point, the liquor which came over was of uniform strength, but, when it rose, the liquor grew weaker and weaker, till at last only water came over,—that is, when the thermometer stood at 80° of Reaumur. His experiments were performed many times, and always with similar results. From these premises he draws the following conclusions:—1. A person may, by the state of the thermometer, immediately ascertain the strength of the liquor in the vessel. 2. There is no necessity of using the *alkohometer* in distillation, as the preceding account shows that the thermometer indicates the strength of the liquor with equal accuracy. 3. Without drawing off any spirit, what quantity there is of any particular strength may be immediately known. 4. Every possible fraud during the operation may be prevented, as the apparatus can either be locked up or brought into an adjoining apartment; for the person who attends the work does not require the thermometer to direct him. It is further stated, that, although the experiments were made with the apparatus of M. Groening, yet he thinks the thermometer, if properly applied to any other, would be attended with similar results. The importance of these statements, both to the chemist and distiller on the great scale, are too obvious to require comment.

The Chromate of Potass has been recommended, by Dr. COOPER, president of Columbia College, as a good test for Arsenic. It is to be used in solution, and one drop is said to be turned green by one-fourth of a grain of arsenic, and by a few drops of the liq. arsenicalis or any other arsenite of potass. The theory is, that the arsenious acid takes away oxygen from the chromic, which is thus converted into a green oxide. To show the experiment, we are directed to take five watch-glasses,—to put into one of them a few drops of a watery solution of white arsenic, into another the arsenite of potass, into a third the fourth of a grain of white arsenic, into the fourth a small quantity of the solution of corrosive sublimate, and into the fifth a little of the solution of copper. To each of these is to be added a few drops of the solution of chromate of potass: in half an hour, the three first will exhibit a bright grass green, which is not changed by ammonia; the fourth will become instantly orange; and the fifth will show a green precipitate, which is converted by a drop of ammonia into blue.*

It is well known that the Liquor Hydrargyri Oxymuriatis of the London Pharmacopœia undergoes decomposition by exposure to light, and it has been said that light produces a similar effect on corrosive sublimate itself. The subject has been inquired into by Dr. DAVY,† who proved that the oxymuriate of mercury remains unaltered on exposure to light in the solid form, and likewise when dissolved in a medium possessing a strong affinity for it,—as alcohol, ether, muriatic acid, &c.; and that decomposition is only effected under the existence of complicated affinities, as in the instance of the liq. hydrargyri oxymuriatis, and in the watery solution when calomel and muriatic acid are formed, and oxygen disengaged. In every instance that an oil, whether fixed or volatile, was heated with corrosive sublimate, mutual decomposition was the result, charcoal was evolved, and muriatic acid and calomel formed. With muriatic acid, common salt, and other muriates, definite compounds were formed, remarkable for their solubility.

The Pulvis Antimonialis of the Lond. Pharm. has likewise been made the subject of experiment by Mr. RICHARD PHILLIP,‡ in consequence of the observations on the doses of that medicine published by Dr. ELLIOTSON, in which cases are related where ninety, and even one hundred, grains were exhibited with safety, and indeed with scarcely any sensible effect. The result of these experiments by Mr. Phillips led him to regard this powder as a mere mixture of metallic oxide with phosphate of lime, in the following proportions:—Peroxide of antimony, 35; phosphate of lime, 65: or, according to the analysis of another specimen, of the former, 38, and of the latter, 62. An opinion nearly similar was given by Mr. CHENEVIX, in the Philosophical Transactions for 1801, to which Mr. Phillips alludes.

### BOTANY.

Several additions have been made to this department of science: these, however, have consisted less in the discovery of any thing new, than in the publication of works tending to give facilities to the student. Among these, the "Lectures on the Elements of Botany," by the accomplished author of the London Dispensatory, deserves particular notice. The first volume only has yet appeared: it contains the descriptive anatomy of those organs on which the growth and preservation of the plant depend, and is accompanied with plates, which illustrate the description in a satisfactory manner. The "Exotic Flora" of Dr. HOOKER likewise promises to be a very valuable addition to the botanical library: it is intended to give figures and descriptions of such plants, not natives of Great Britain, as are cultivated in gardens; or, in defect of these, of such as can be faithfully represented from well-preserved specimens in the Herbaria. Another work has likewise been commenced by Mr. GREVILLE, on the Cryptogamia, of Scotland, with coloured plates, in the manner of the "English Botany," by SMITH and SOWERBY.—This last work only wants the fungi to render it complete.

* SILLIMAN'S *Journal*. † *Ann. Philosophy*, July. ‡ *Ib.* October.

